# Endoplasmic reticulum stress inhibition ameliorated WFS1 expression alterations and reduced pancreatic islets’ insulin secretion induced by high-fat diet in rats

**DOI:** 10.1038/s41598-023-28329-1

**Published:** 2023-02-01

**Authors:** Fateme Binayi, Javad Fahanik-Babaei, Mina Salimi, Farzaneh Eskandari, Mohammad Sahraei, Ali Ghorbani Ranjbary, Rasoul Ghasemi, Mehdi Hedayati, Fariba Khodagholi, Afsaneh Eliassi, Homeira Zardooz

**Affiliations:** 1 Neurophysiology Research Center, Shahid Beheshti University of Medical Sciences, Tehran, Iran; 2 Department of Physiology, School of Medicine, Shahid Beheshti University of Medical Sciences, Tehran, Iran; 3 Electrophysiology Research Center, Tehran University of Medical Science, Tehran, Iran; 4 School of Dentistry, Shahid Beheshti University of Medical Sciences, Tehran, Iran; 5 Department of Microbiology and Immunology, Faculty of Veterinary Medicine, University of Tehran, Tehran, Iran; 6 Cellular and Molecular Endocrine Research Center, Research Institute for Endocrine Sciences, Shahid Beheshti University of Medical Sciences, Tehran, Iran; 7 Neuroscience Research Center, Shahid Beheshti University of Medical Sciences, Tehran, Iran

**Keywords:** Biochemistry, Physiology, Endocrinology

## Abstract

Endoplasmic reticulum (ER) stress is involved in the development of glucose homeostasis impairment. When ER stress occurs, the unfolded protein response (UPR) is activated to cope with it. One of the UPR components is WFS1 (Wolfram syndrome 1), which plays important roles in ER homeostasis and pancreatic islets glucose-stimulated insulin secretion (GSIS). Accordingly and considering that feeding high-fat food has a major contribution in metabolic disorders, this study aimed to investigate the possible involvement of pancreatic ER stress in glucose metabolism impairment induced by feeding high-fat diet (HFD) in male rats. After weaning, the rats were divided into six groups, and fed on normal diet and HFD for 20 weeks, then 4-phenyl butyric acid (4-PBA, an ER stress inhibitor) was administered. Subsequently, in all groups, after performing glucose tolerance test, the animals were dissected and their pancreases were removed to extract ER, islets isolation and assessment of GSIS. Moreover, the pancreatic ER stress [binding of immunoglobulin protein (BIP) and enhancer-binding protein homologous protein (CHOP)] and oxidative stress [malondialdehyde (MDA), glutathione (GSH) and catalase] biomarkers as well as WFS1 expression level were evaluated. HFD decreased pancreatic WFS1 protein and GSH levels, and enhanced pancreatic catalase activity, MDA content, BIP and CHOP protein and mRNA levels as well as *Wfs1* mRNA amount. Accordingly, it increased BIP, CHOP and WFS1 protein levels in the extracted ER of pancreas. In addition, the HFD caused glucose intolerance, and decreased the islets’ GSIS and insulin content. However, 4-PBA administration restored the alterations. It seems that, HFD consumption through inducing pancreatic ER stress, altered WFS1 expression levels, reduced the islets’ GSIS and insulin content and finally impaired glucose homeostasis.

## Introduction

High-fat diet (HFD) consumption causes various metabolic diseases, including metabolic syndrome and type 2 diabetes by developing insulin resistance and subsequently decreasing insulin production^1–3^. Therefore, it appears that a common characteristic of most metabolic diseases induced by HFD is the reduction of insulin synthesis and secretion from pancreatic islets^4^. Long-term exposure to saturated fatty acids (SFA) in pancreatic β cells causes desensitization and impaired insulin secretion^5^. In this regard, a 48-h infusion of palmitate (SFA) reduced both primary and secondary phases of insulin secretion, with more profound impact as the exposure period was longer^6^. Furthermore, numerous investigations have demonstrated that long-term HFD feeding decreases pancreatic islets insulin synthesis and content^7–9^. For example, three-month consumption of HFD containing pork fat and sunflower oil (80% fat) reduced the pancreatic islets insulin content (50%), proinsulin mRNA (35%), insulin biosynthesis and secretion in response to glucose (50%), and glucose oxidation in mice^10^. According to previous research, WFS1 (Wolfram syndrome 1) is involved in the synthesis and release of insulin, as well as the preservation of the pancreatic β cell mass^11–14^. *Wfs1* gene was first identified by Wolfram and Wagener (1983) in patients with Wolfram syndrome (i.e. diabetes mellitus and optic nerve atrophy)^12,15,16^. WFS1 is a glycoprotein expressed in the endoplasmic reticulum (ER) of pancreatic β cells, heart, placenta, lung, and brain^15–17^. It is worth noting that the β cells of endocrine pancreas are the major site of WFS1 expression and no signs of its expression has been found in exocrine pancreas^18^. Fonseca et al. showed that WFS1 played a crucial role in calcium-dependent insulin synthesis and secretion in response to increased glucose concentration^12^. WFS1, as one of unfolded protein response (UPR)'s downstream components, is involved in the maintenance of ER homeostasis^19,20^. During ER stress, the WFS1 expression rises to inhibit ER stress signaling and to prevent apoptosis^20^. Several studies have demonstrated that feeding a HFD causes oxidative damage^21–23^, which could be attributed to an increase in corticosterone concentration^24^ due to elevated activity of the hypothalamic–pituitary–adrenal (HPA) axis^25–27^ or an increase in the free fatty acid (FFA) concentration^28^. Moreover, SFAs like palmitate induced ER stress by activating inositol-requiring enzyme1 (IRE1), protein kinase RNA-like endoplasmic reticulum kinase (PERK), and activating transcription factor 6 (ATF6) pathways (UPR-related pathways)^29–31^. Choi et al. discovered that application of palmitate in the INS-1 cells (β cells) culture medium induced ER stress and decreased glucose-stimulated insulin secretion (GSIS). Studies have shown that there is a mutual relationship between oxidative stress and ER stress. It is revealed that the use of chemical chaperone 4-phenyl butyric acid (4-PBA), inhibited ER stress and improved GSIS^32^. According to the abovementioned findings, it can be postulated that HFD causes upregulation of the *Wfs1* mRNA level in β cells of pancreatic islets by inducing ER stress. Furthermore, given the role of the WFS1 protein in maintaining ER homeostasis, the expression of this protein is expected to be increased in β cells’ ER, and its translocation to the cytoplasm is reduced and led to a decrease in the pancreatic islets’ GSIS and insulin content. Although numerous studies have investigated the effects of each HFD and the involvement of WFS1 in insulin synthesis and secretion, no studies have examined the interaction of HFD and WFS1 in relation to insulin synthesis and secretion and hence glucose homeostasis. Considering that WFS1 is expressed mainly in β cells, not in other pancreatic cells^12^, in the current study, the effect of long-term HFD (31% by weight of cow butter) on the induction of pancreatic oxidative stress, ER stress, and WFS1 expression in relation with glucose-stimulated insulin secretion and content of pancreatic isolated islets were investigated in adult male Wistar rats. To confirm the mentioned hypothesis, 4-PBA (an ER stress inhibitor) was administered. The animal species and the dietary fat composition used in this study, could provide an animal model which closely mimic the level and composition of fat in the human diet and could develop obesity and pathologies such as diabetes and metabolic disorder^33,34^.

## Results

### The effect of high-fat diet and/or 4-PBA on pancreatic malondialdehyde (MDA) and glutathione (GSH) levels and total catalase activity

**Figure 1:**
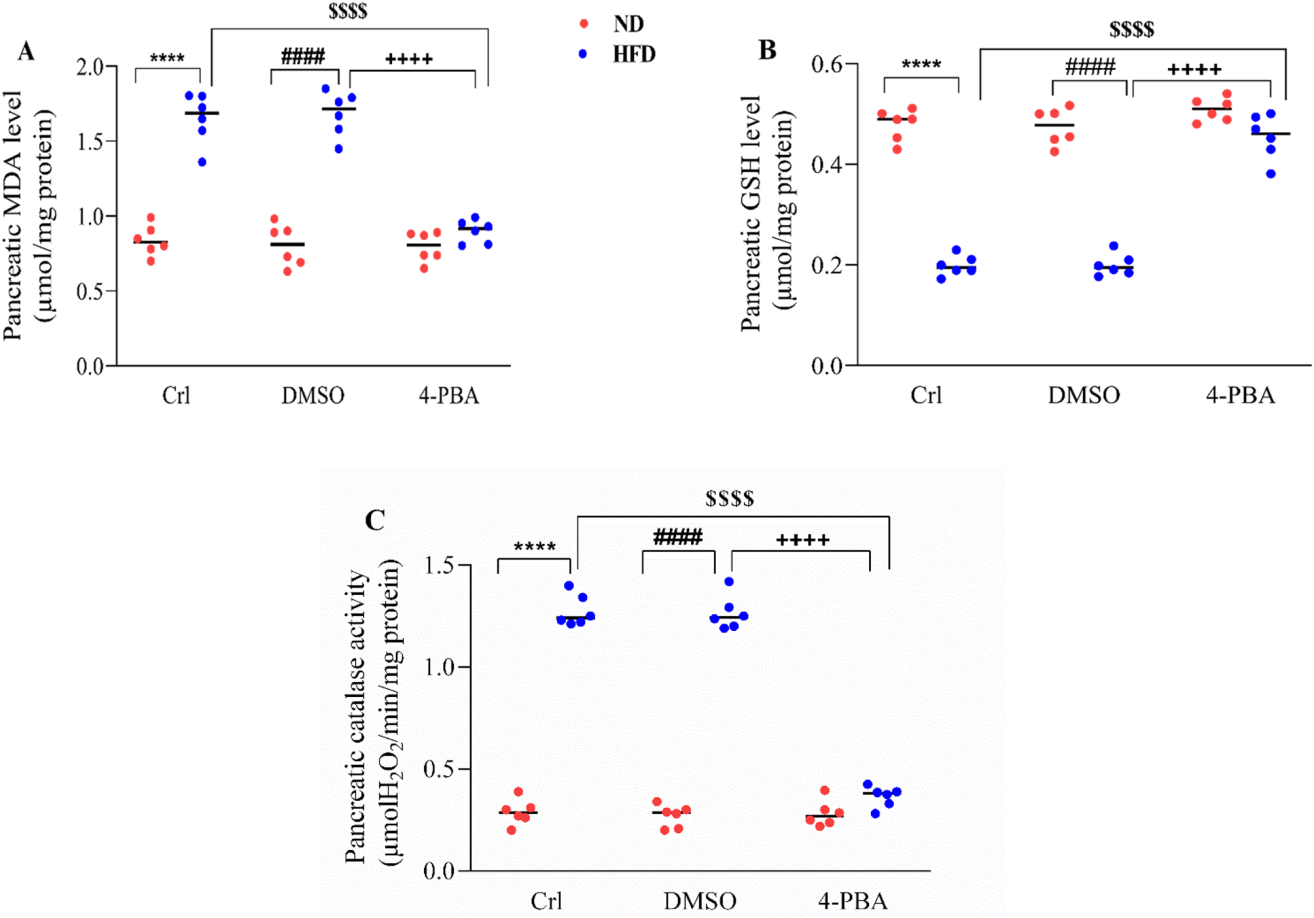
Effect of high-fat diet and/or 4-PBA on pancreatic (**A**) MDA and (**B**) GSH levels and (**C**) total catalase activity. Each point represents the mean ± SEM (6 rats/group). ****p < 0.0001 versus control of ND group, ^####^p < 0.0001 versus ND + DMSO group, ^$$$$^p < 0.0001 versus control of HFD group, ^++++^p < 0.0001 versus HFD + DMSO group. *ND* Normal diet, *HFD* High-fat diet, *Crl* Control, *DMSO* Dimethyl Sulfoxide, *4-PBA* 4-Phenyl Butyric Acid.

HFD consumption significantly increased the pancreatic MDA level and decreased its GSH content in HFD and HFD+ dimethyl sulfoxide (DMSO) groups, compared to ND and ND+ DMSO groups (P < 0.0001) (Fig. 1A,B). The injection of 4-PBA in the HFD + 4-PBA group significantly increased the GSH and decreased MDA levels of the pancreas compared to HFD and HFD + DMSO groups (P < 0.0001) (Fig. 1A,B), so that there was no significant difference compared to the ND groups (Fig. 1A,B). Compared to the control group, the DMSO injection did not significantly change the pancreatic GSH and MDA content in any of the diet groups (Fig. 1A,B).

Compared to ND and ND + DMSO groups, the catalase activity in the pancreas of HFD and HFD + DMSO groups increased considerably following the HFD feeding (P < 0.0001) (Fig. 1C). In the HFD + 4-PBA group, injection of 4-PBA significantly decreased the activity of this enzyme compared to HFD and HFD + DMSO groups (P < 0.0001) (Fig. 1C); however, there were no significant difference compared to the respective ND groups (Fig. 1C). In any of diet groups, the DMSO injection had no effect on the catalase activity, when compared to the control groups (Fig. 1C).

### The effect of high-fat diet and/or 4-PBA on pancreatic C/EBP-homologous protein *(Chop)*, immunoglobulin heavy chain binding protein* (Bip)* and *Wfs1* mRNA levels

**Figure 2:**
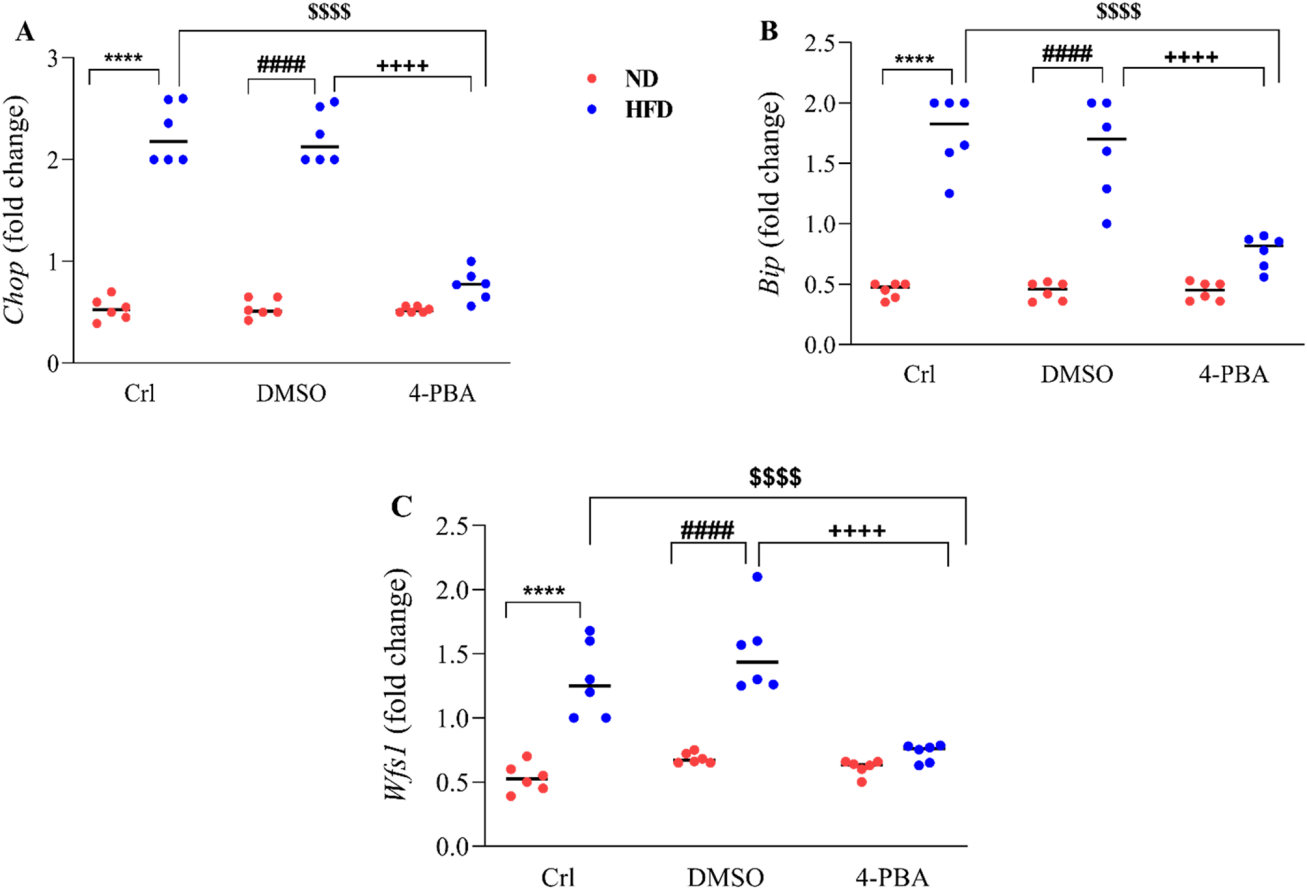
Effect of high-fat diet and/or 4-PBA on pancreatic (**A**) *Chop*, (**B**) *Bip* and (**C**) *Wfs*1 mRNA levels. Each point represents the mean ± SEM (6 rats/group). ****p < 0.0001 versus control of ND group, ^####^p < 0.0001 versus ND + DMSO group, ^$$$$^p < 0.0001 versus control of HFD group, ^++++^p < 0.0001 versus HFD + DMSO group. *ND* Normal diet, *HFD* High-fat diet, *Crl* Control, *DMSO* Dimethyl Sulfoxide, *4-PBA* 4-Phenyl Butyric Acid.

HFD feeding caused a substantial increase in *Chop* (Fig. 2A), *Bip* (Fig. 2B) and *Wfs1* (Fig. 2C) mRNA levels in the pancreas of HFD and HFD + DMSO groups compared to ND and ND + DMSO groups (P < 0.0001). Administration of 4-PBA reduced the expression of these genes in the HFD + 4-PBA group than in HFD and HFD + DMSO groups (P < 0.0001), so that there was no significant difference compared to the ND groups (Fig. 2A–C). Injection of DMSO had no effect on the expression of these genes in any of the diet groups (Fig. 2A–C).

### The effect of high-fat diet and/or 4-PBA on pancreatic CHOP, BIP and WFS1 protein levels

**Figure 3:**
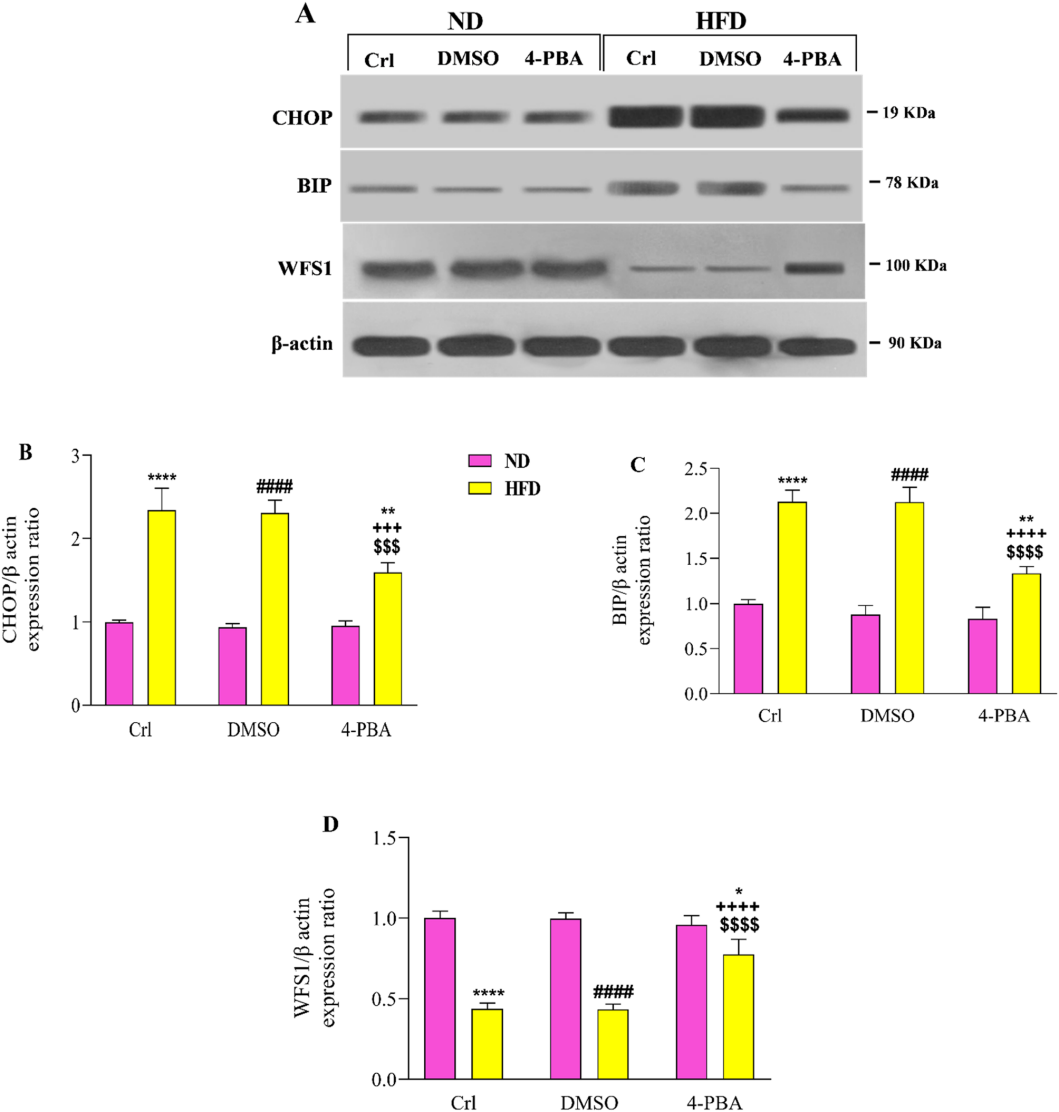
Effect of high-fat diet and/or 4-PBA on the (**A**,**B**) CHOP, (**A**,**C**) BIP and (**A**,**D**) WFS1 protein levels of the pancreas. Each column represents the mean ± SEM (10 rats/group). *p < 0.05, **p < 0.01, ****p < 0.0001 versus control of ND group, ^####^p < 0.0001 versus ND + DMSO group, ^$$$^p < 0.001, ^$$$$^p < 0.0001 versus control of HFD group, ^+++^p < 0.001, ^++++^p < 0.0001 versus HFD + DMSO group. *ND* Normal diet, *HFD* High-fat diet, *Crl* Control, *DMSO* Dimethyl Sulfoxide, *4-PBA* 4-Phenyl Butyric Acid.

Compared to ND and ND + DMSO groups, HFD caused a substantial increase in pancreatic CHOP (Fig. 3A,B) and BIP (Fig. 3A,C) protein levels in HFD and HFD + DMSO groups (P < 0.0001). The levels of CHOP and BIP proteins in the pancreas were considerably lower in the HFD + 4-PBA group than in HFD (P < 0.001, P < 0.0001) and HFD + DMSO (P < 0.001, P < 0.0001) groups; however, these values were significantly higher than in the ND groups (P < 0.01) (Fig. 3A–C). Compared to the control group, DMSO injection had no effect on the levels of these proteins in any of the diet groups (Fig. 3A–C).

HFD consumption significantly reduced the pancreatic WFS1 protein level in HFD and HFD + DMSO groups compared to ND and ND + DMSO groups (P < 0.0001) (Fig. 3A,D). Administration of 4-PBA in the HFD + 4-PBA group increased the WFS1 protein expression in the pancreas compared to HFD and HFD + DMSO groups (P < 0.0001); however, its value was significantly lower than that in the ND groups (P < 0.05) (Fig. 3A,D). Compared to the control group, DMSO injection had no effect on the level of this protein in any of the diet groups (Fig. 3A,D).

### The effect of high-fat diet and/or 4-PBA on the protein levels of CHOP, BIP and WFS1 in the extracted ER of pancreas

**Figure 4:**
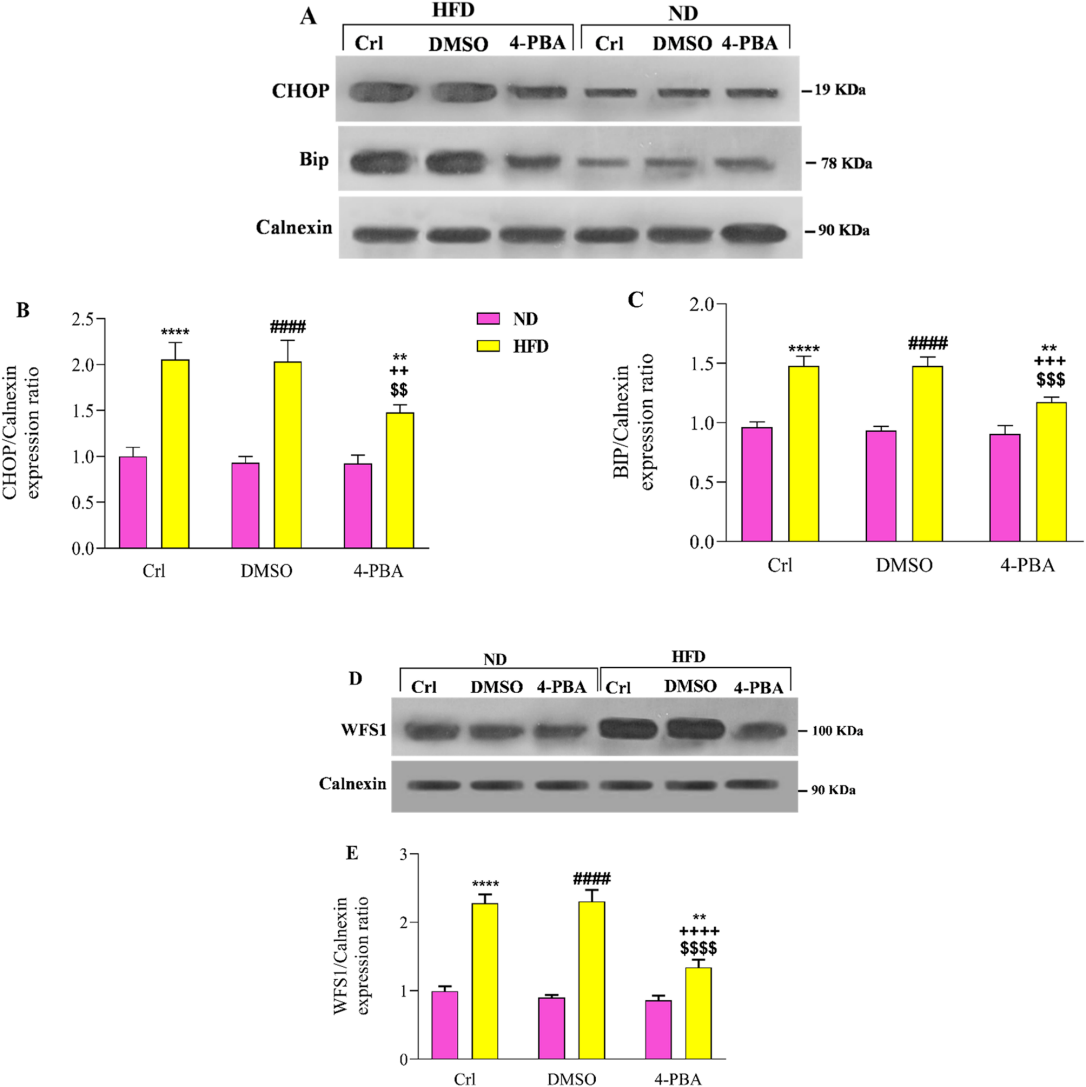
Effect of high-fat diet and/or 4-PBA on the (**A**,**B**) CHOP, (**A**,**C**) BIP and (**D**,**E**) WFS1 protein levels of the extracted ER of pancreas. Each column represents the mean ± SEM (10 rats/group). **p < 0.01, ****p < 0.0001 versus control of ND group, ^####^p < 0.0001 versus ND + DMSO group, ^$$^p < 0.01, ^$$$^p < 0.001, ^$$$$^p < 0.0001 versus control of HFD group, ^++^p < 0.01, ^+++^p < 0.001, ^++++^p < 0.0001 versus HFD + DMSO group. *ND* Normal diet, *HFD* High-fat diet, *Crl* Control, *DMSO* Dimethyl Sulfoxide, *4-PBA* 4-Phenyl Butyric Acid.

According to the statistical analysis, consumption of HFD substantially increased the protein levels of CHOP (Fig. 4A,B) and BIP (Fig. 4A,C) in the extracted ER of the pancreas in HFD and HFD + DMSO groups, compared to ND and ND + DMSO groups (P < 0.0001). In comparison to HFD and HFD + DMSO groups, 4-PBA injection reduced the protein levels of CHOP (P < 0.01) and BIP (P < 0.001) in the pancreatic extracted ER; however, these values were significantly higher than in the ND groups (P < 0.01) (Fig. 4A–C). In comparison to the control group, DMSO injection had no effect on the levels of these proteins in any of the diet groups (Fig. 4A–C).

Compared to ND and ND + DMSO groups, there was a substantial increase in the WFS1 protein level in the pancreatic extracted ER of HFD and HFD + DMSO groups (P < 0.0001) (Fig. 4D,E). Administration of 4-PBA in the HFD + 4-PBA group decreased the WFS1 protein level in the extracted ER compared to HFD and HFD + DMSO groups (P < 0.0001) (Fig. 4D,E); however, its value was significantly higher than that of the ND groups (P < 0.01) (Fig. 4D,E). In comparison to the control group, DMSO injection had no effect on the level of this protein in any of the diet groups (Fig. 4D,E).

### The effect of high fat diet and/or 4-PBA on the pancreatic isolated islets’ insulin secretion and insulin content in response to glucose

**Figure 5:**
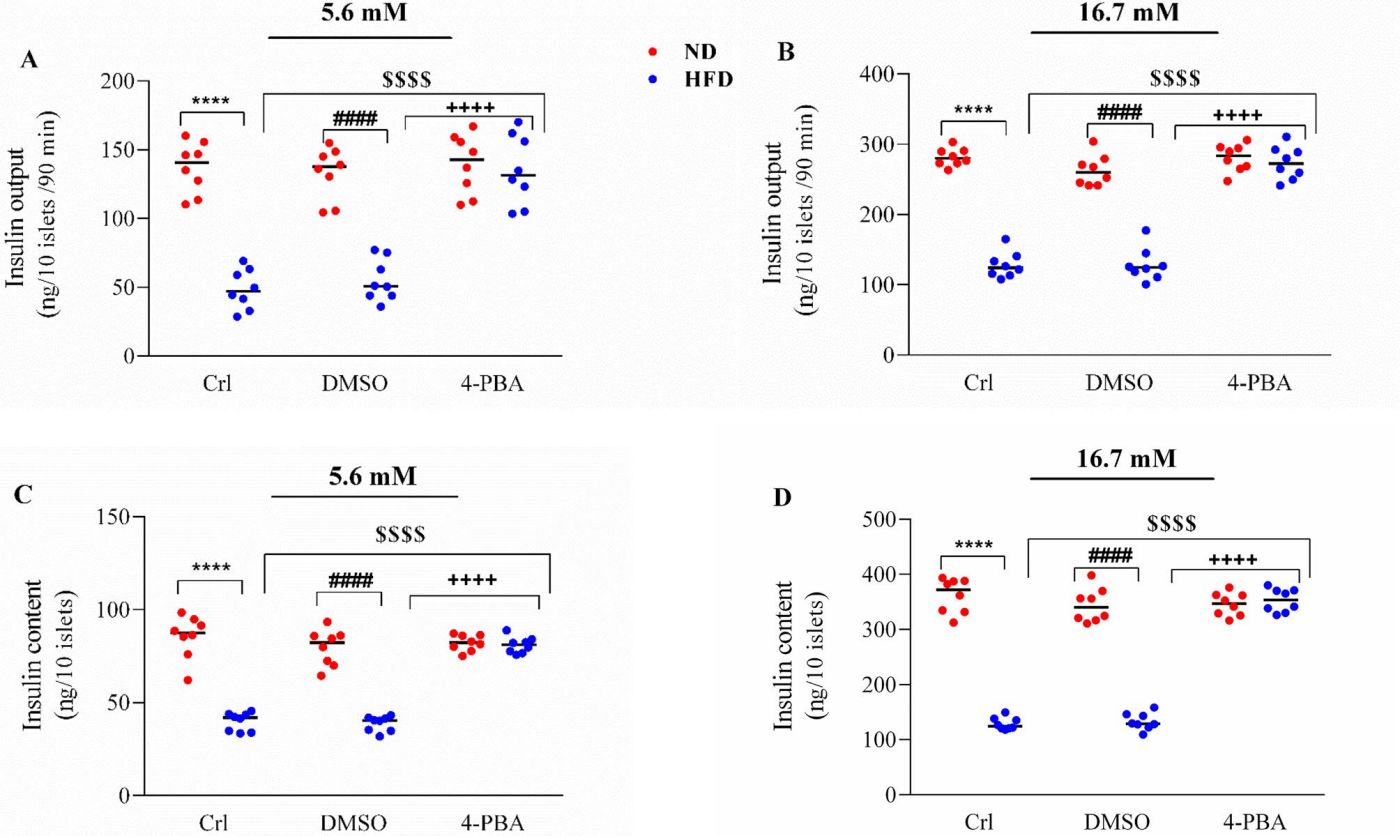
Effect of high-fat diet and/or 4-PBA on the pancreatic isolated islets’ (**A**,**B**) insulin output and (**C**,**D**) insulin content in the presence of 5.6 and 16.7 mM glucose concentrations, respectively. Each point represents the mean ± SEM (4 rats/group). ****p < 0.0001 versus control of ND group, ^####^p < 0.0001 versus ND + DMSO group, ^$$$$^p < 0.0001 versus control of HFD group, ^++++^p < 0.0001 versus HFD + DMSO group. *ND* Normal diet, *HFD* High-fat diet, *Crl* Control, *DMSO* Dimethyl Sulfoxide, *4-PBA* 4-Phenyl Butyric Acid.

Consumption of HFD significantly decreased the pancreatic isolated islets’ insulin secretion in response to 5.6 and 16.7 mM glucose concentrations in HFD and HFD + DMSO groups, compared to ND and ND + DMSO groups (P < 0.0001) (Fig. 5A,B). In the presence of both glucose concentrations, the 4-PBA injection caused a significant increase of insulin secretion in the HFD + 4-PBA group, compared to HFD and HFD + DMSO groups (P < 0.0001), so that its value did not show any significant difference compared to the ND groups (Fig. 5A,B). Injection of DMSO had no effect on the insulin secretion in any of the diet groups (Fig. 5A,B).

Compared to ND and ND + DMSO groups, HFD and HFD + DMSO groups had significantly lower pancreatic islets’ insulin content in response to 5.6 and 16.7 mM glucose concentrations (P < 0.0001) (Fig. 5C,D). Injection of 4-PBA significantly enhanced the insulin content of the HFD + 4-PBA group in response to both glucose concentrations, compared to HFD and HFD + DMSO groups (P < 0.0001), so that, there was no significant difference compared to the ND groups (Fig. 5C,D). Injection of DMSO had no effect on the insulin content in any of the diet groups (Fig. 5C,D).

### The effect of high-fat diet and/or 4-PBA on fasting plasma glucose and insulin levels and HOMA-β%

**Figure 6:**
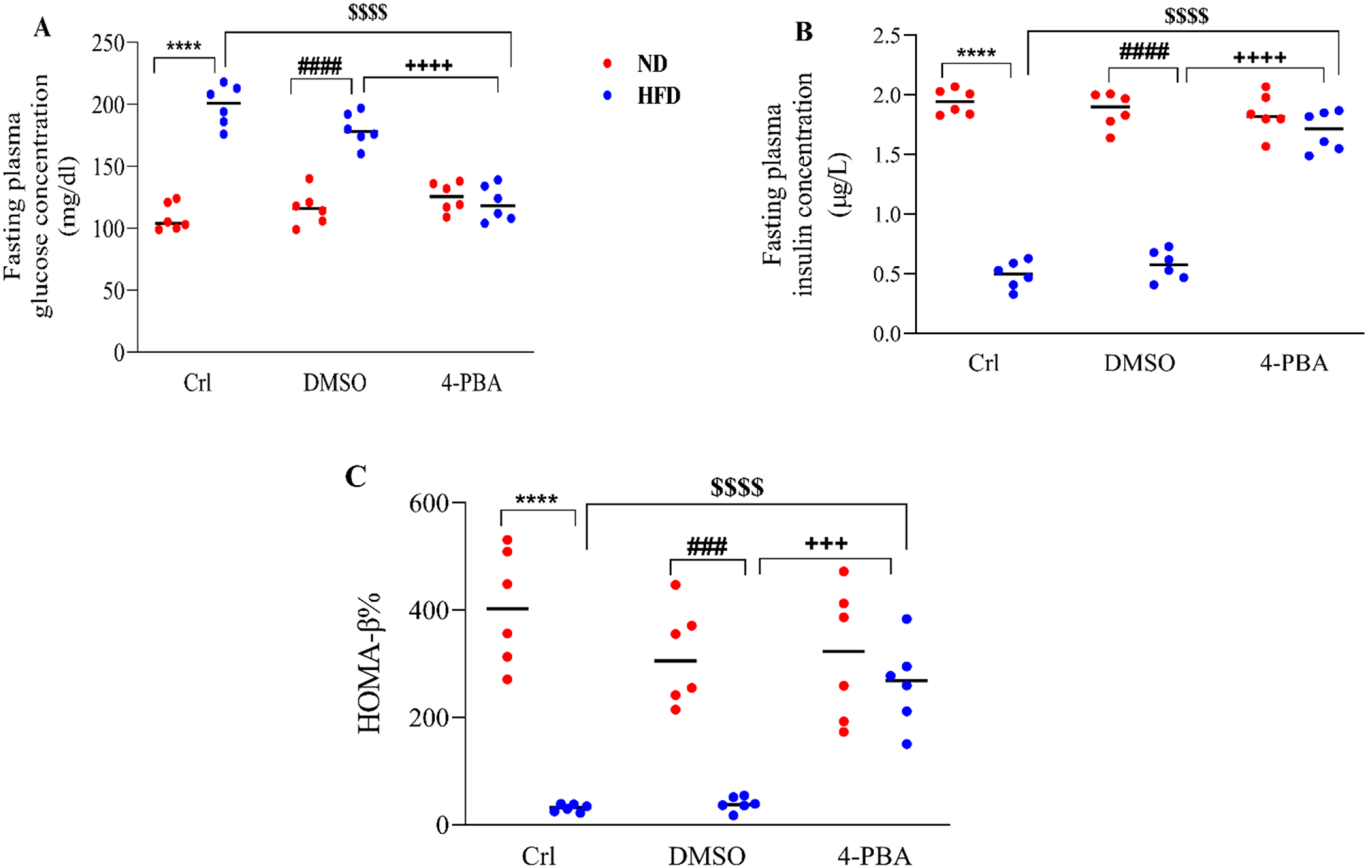
Effect of high-fat diet and/or 4-PBA on fasting plasma (**A**) glucose and (**B**) insulin levels and (**C**) HOMA-β%. Each point represents the mean ± SEM (6 rats/group). ****p < 0.0001 versus control of ND group, ^###^p < 0.001, ^####^p < 0.0001 versus ND + DMSO group, ^$$$$^p < 0.0001 versus control of HFD group, ^+++^p < 0.001, ^++++^p < 0.0001 versus HFD + DMSO group. *ND* Normal diet, *HFD* High-fat diet, *Crl* Control, *DMSO* Dimethyl Sulfoxide, *4-PBA* 4-Phenyl Butyric Acid.

Compared to the ND and ND + DMSO groups, the HFD induced a substantial rise in fasting plasma glucose (P < 0.0001) (Fig. 6A), and a significant reduction in fasting plasma insulin (P < 0.0001) (Fig. 6B). Moreover, HFD significantly decreased HOMA**-**β% (P < 0.0001, P<0.001) (Fig. 6C) index compared to the ND and ND + DMSO groups. Injection of 4-PBA, decreased the fasting plasma glucose concentration (P < 0.0001) (Fig. 6A), increased the fasting plasma insulin concentration (P < 0.0001) (Fig. 6B), and HOMA-β% (P < 0.0001, P < 0.001) (Fig. 6C) index in the HFD + 4-PBA group compared to HFD and HFD + DMSO groups, so that, there were no significant difference compared to normal diet groups (Fig. 6A–C). Moreover, DMSO injection did not change the mentioned parameters compared to the control groups of any diet groups (Fig. 6A–C).

### The effect of high-fat diet and/or 4-PBA on plasma glucose and insulin concentrations during oral glucose tolerance test (OGTT)

**Figure 7:**
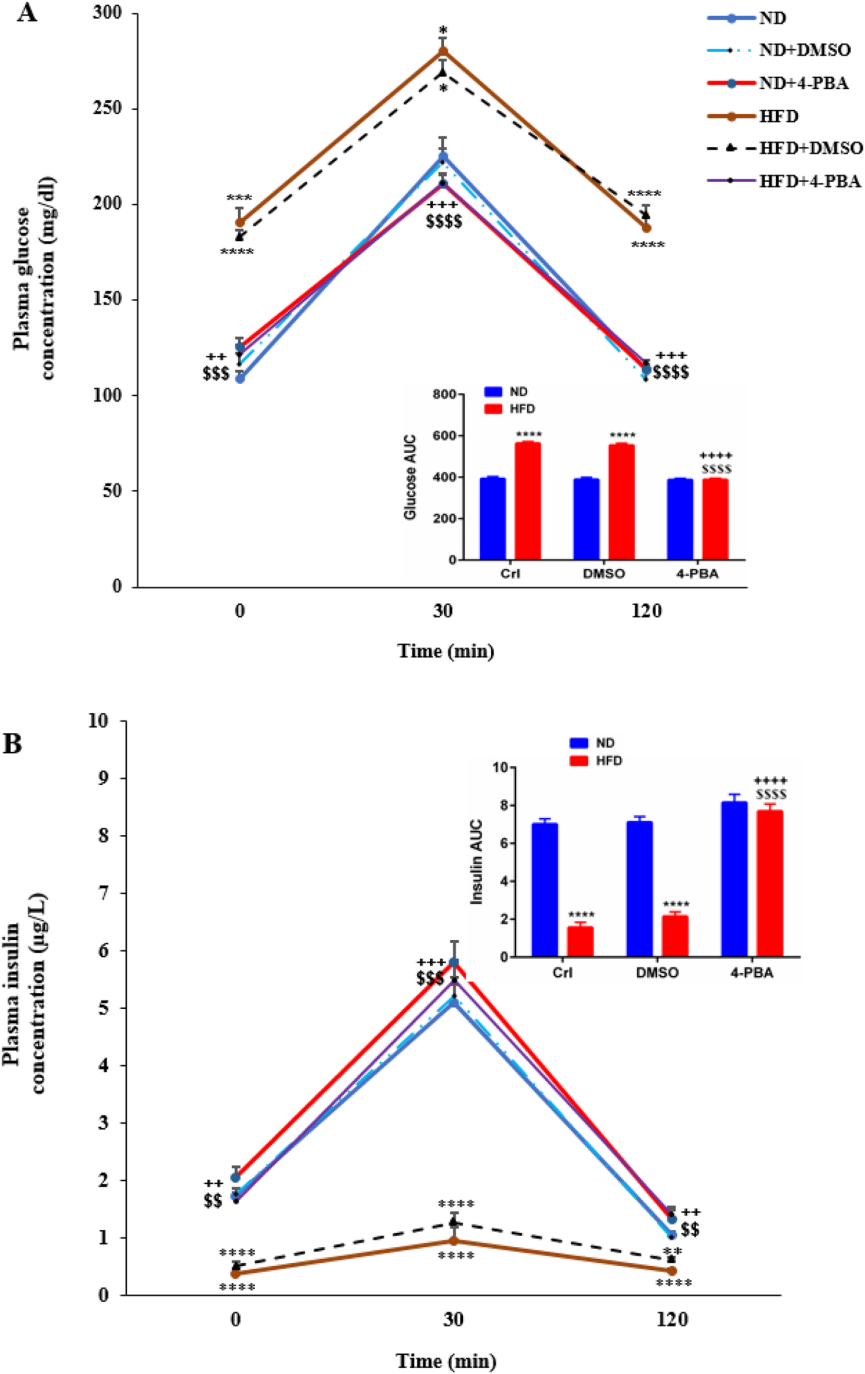
Effect of high-fat diet and/or 4-PBA on plasma (**A**) glucose and (**B**) insulin concentrations during OGTT. Each point/column represents mean ± SEM (6 rats/group). *p<0.05, **p<0.01, ***p < 0.001, ****p < 0.0001 versus ND group, ^$$^p < 0.01, ^$$$^p < 0.001, ^$$$$^p < 0.0001 versus HFD group, ^++^p < 0.01, ^+++^p < 0.001 ^++++^p < 0.0001versus HFD + DMSO group, at the same time. *ND* Normal diet, *HFD* High-fat diet, *Crl* Control, *DMSO* Dimethyl Sulfoxide, *4-PBA* 4-PhenylButyric Acid, *AUC* area under the curve.

The OGTT curve exhibited a substantial rise in the plasma glucose concentration in the control (P < 0.001) and DMSO (P < 0.0001) groups of HFD at 0 min compared to the respective ND groups (Fig. 7A). In addition, 30 min after glucose gavage, the plasma glucose concentrations in HFD and HFD + DMSO groups were 280.5 ± 6.2 mg/dl and 268.67 ± 7 mg/dl, respectively, being significantly higher than that in ND (225.3 ± 9.9 mg/dl) group (P < 0.05) (Fig. 7A). Furthermore, at 120 min, the plasma glucose concentrations in HFD and HFD + DMSO groups were substantially higher than that in ND group (P < 0.0001) (Fig. 7A). 4-PBA injection caused a significant decrease in this parameter at all three test times in HFD + 4-PBA group compared to that in HFD and HFD + DMSO groups (0 min (P < 0.001, P < 0.01), 30 min (P < 0.0001, P < 0.001) and 120 min (P < 0.0001, P < 0.001), respectively) (Fig. 7A); so that this group did not show significant difference with ND and ND + DMSO groups at any time point (Fig. 7A). The area under the curve (AUC) of plasma glucose changes during OGTT confirmed the above mentioned explanations (inset of Fig. 7A).

Moreover, the OGTT curve showed a substantial reduction in the plasma insulin concentration at 0 min in the control and DMSO groups of HFD compared to the respective ND groups (P < 0.0001) (Fig. 7B). The plasma insulin concentration in HFD and HFD + DMSO groups 30 min after glucose gavage was 0.95 ± 0.23 µg/L and 1.27 ± 0.17 µg/L, respectively, being significantly lower than that in ND (5.09 ± 0.3 µg/L) (P < 0.0001) (Fig. 7B) groups. HFD (P < 0.0001) and HFD + DMSO (P < 0.01) groups had substantially lower plasma insulin concentrations at 120 min than ND group (Fig. 7B). This parameter significantly increased after 4-PBA injection at all three test times in the HFD + 4-PBA group compared to that in HFD and HFD + DMSO groups (0 min (P < 0.01), 30 min (P < 0.001) and 120 min (P < 0.01), respectively) (Fig. 7B), so that this group did not show significant difference compared to ND and ND + DMSO groups at any time point (Fig. 7B). The area under the curve (AUC) of plasma insulin changes during OGTT confirmed the above mentioned explanations (inset of Fig. 7B).

### The effect of high-fat diet and/or 4-PBA on plasma corticosterone and leptin concentrations

**Figure 8:**
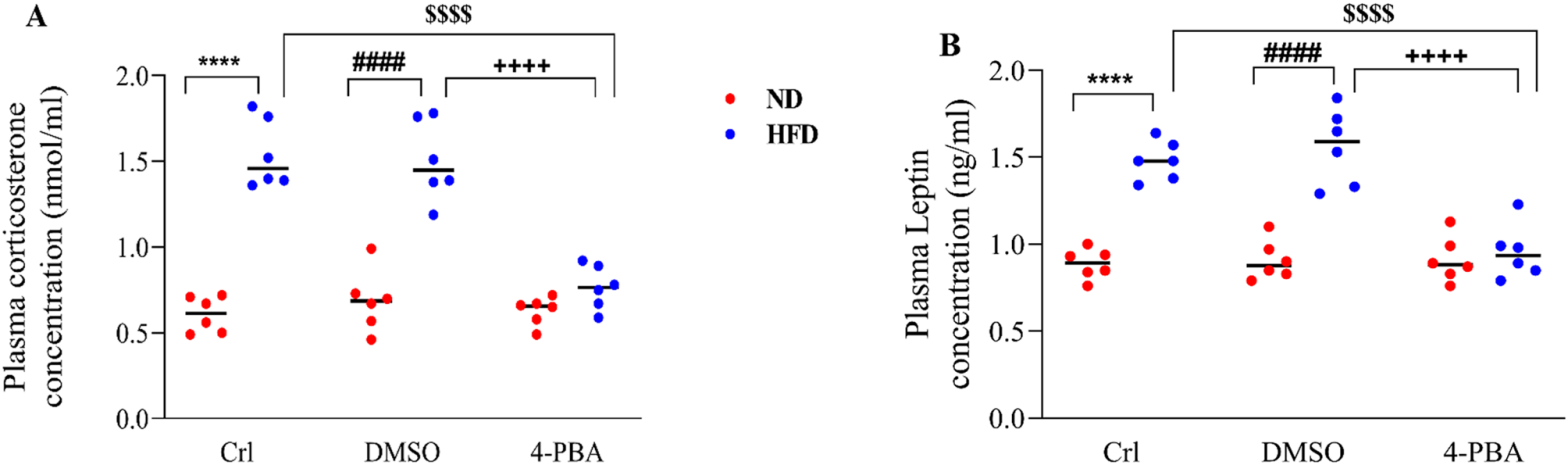
Effect of high-fat diet and/or 4-PBA on plasma (**A**) Corticosterone and (**B**) Leptin concentrations. Each point represents the mean ± SEM (6 rats/group). ****p < 0.0001 versus control of ND group, ^####^p < 0.0001 versus ND + DMSO group, ^$$$$^p < 0.0001 versus control of HFD group, ^++++^p < 0.0001 versus HFD + DMSO group. *ND* Normal diet, *HFD* High-fat diet, *Crl* Control, *DMSO* Dimethyl Sulfoxide, *4-PBA* 4-Phenyl Butyric Acid.

When comparing HFD and HFD + DMSO groups to ND and ND + DMSO groups, plasma corticosterone and leptin concentrations were substantially higher in HFD and HFD + DMSO groups (P < 0.0001) (Fig. 8A,B). The 4-PBA injection in the HFD + 4-PBA group resulted in a substantial reduction in these parameters, when compared to HFD and HFD + DMSO groups (P < 0.0001) (Fig. 8A,B), so that there were no significant difference compared to ND groups (Fig. 8A,B). On the contrary, DMSO administration had no effect on plasma corticosterone and leptin levels, when compared to the control groups (Fig. 8A,B).

### The effect of high-fat diet and/or 4-PBA on the weight of abdominal, mesenteric, retroperitoneal fats and body organs

**Figure 9:**
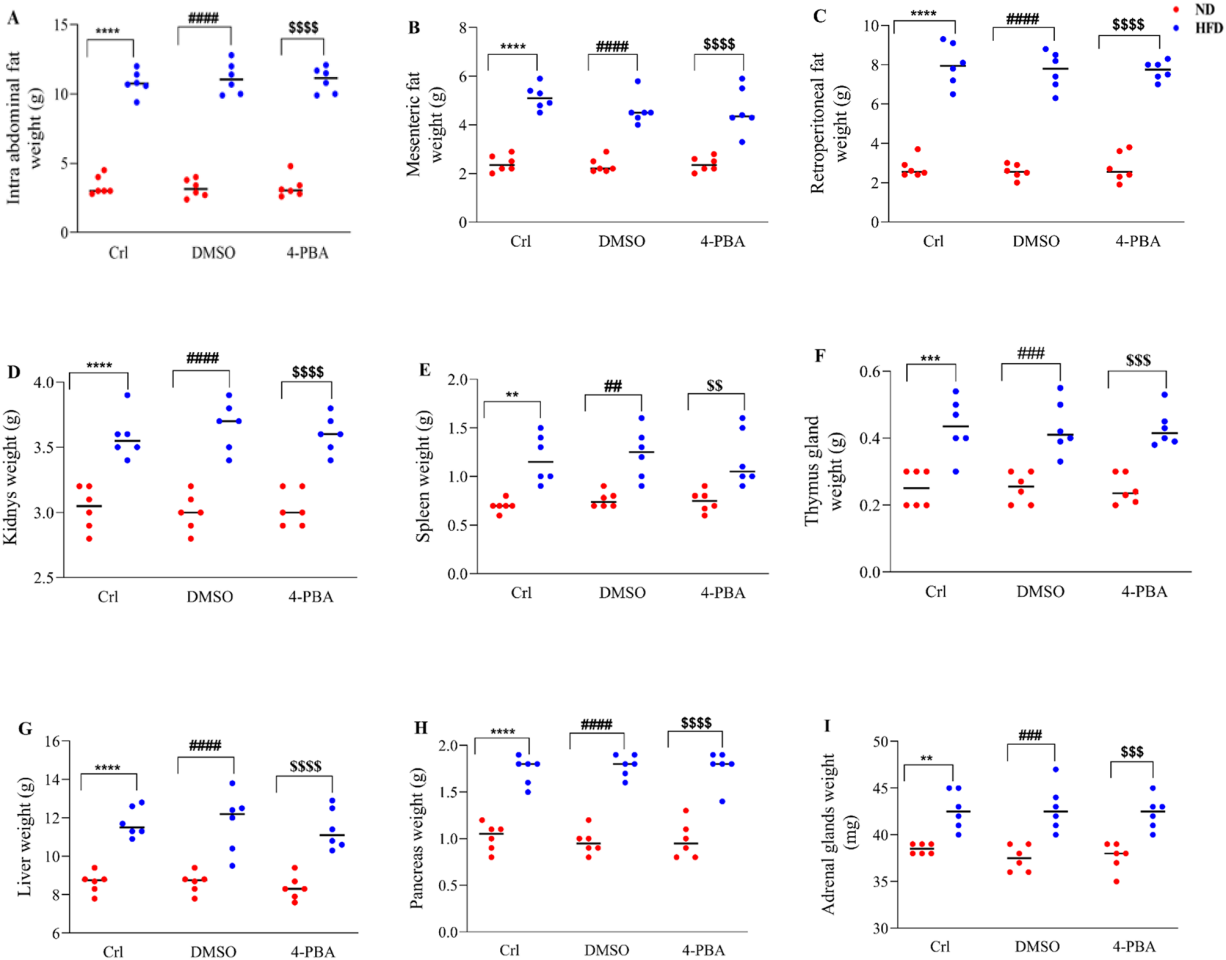
Effect of high-fat diet and/or 4-PBA on the weight of (**A**) intraabdominal, (**B**) mesenteric and (**C**) retroperitoneal fats, and (**D**) kidneys, (**E**) spleen, (**F**) thymus gland, (**G**) liver, (**H**) pancreas and (**I**) adrenal glands. Each point represents the mean ± SEM (6 rats/group). **p < 0.01, ***p < 0.001, ****p < 0.0001, versus control of ND group, ^##^p < 0.01, ^###^p < 0.001, ^####^p < 0.0001 versus ND + DMSO group, ^$$^p < 0.01, ^$$$^p < 0.001, ^$$$$^p < 0.0001 versus ND + 4-PBA. *ND* Normal diet, *HFD* High-fat diet, *Crl* Control, *DMSO* Dimethyl Sulphoxide, *4-PBA* 4-Phenyl Butyric Acid.

Two way analysis of variance revealed that the HFD group had significantly more intra abdominal, mesenteric, and retroperitoneal fat (Fig. 9A–C) than the ND group (P < 0.0001). As 4-PBA and DMSO were injected into HFD and ND groups, no significant changes in intraabdominal, mesenteric, or retroperitoneal fat, were observed when compared to the control groups (Fig. 9A–C). The weight of the kidneys, spleen, thymus, liver, pancreas, and adrenal glands increased significantly (P < 0.0001) in the HFD group than in the ND group, the injection of 4-PBA medication in the HFD + 4-PBA group had no influence on the weight of these organs (Fig. 9D–I). Furthermore, as compared to the control group, the DMSO injection did not induce any significant change in the weight of these organs (Fig. 9D–I).

## Discussion

Chronic HFD increased pancreatic oxidative stress and ER stress biomarkers, as well as *Wfs1* mRNA level in HFD fed animals*.* It is noteworthy that in these animals, although the protein level of WFS1 in the extracted ER of pancreas was increased, its level in the pancreas tissue was significantly decreased. The alteration of the indicated variables restored in HFD rats following the administration of 4-PBA.

The HFD consumption enhanced the pancreatic MDA content and activity of catalase, while decreased its GSH level. High-Fat feeding also increased the *Chop* and *Bip* mRNA and protein levels, as ER stress markers, in the pancreatic tissue and extracted ER. These alterations were considerably improved, when 4-PBA was administered. In line with the present study results, consumption of HFDs containing 60% lard for 8 weeks^35^ and 49% beef tallow for 8 weeks^36^ increased the MDA content and reduced the GSH level, in the rat pancreatic and liver tissues respectively, indicating the presence of oxidative stress. In the current study, increased catalase activity also suggested oxidative stress in the pancreatic tissue. In agreement with the present study results, 70-day consumption of HFD containing 34.9% lard enhanced the catalase activity in the rat retroperitoneal adipose tissue, being associated with elevated MDA^37^. Moreover, studies have revealed that a persistent rise in blood glucose, as observed in the present study, increases the demand for insulin, which can be resulted in pancreatic ER stress^38^. In this regard, our results showed that chronic HFD increased the expression of *Bip* and *Chop* mRNA and protein in pancreas, suggesting that ER stress was induced. In consistent with our findings, several studies have found that HFD induces ER stress^39–42^. In addition, incubation of isolated Langerhans islets or beta cells with SFAs resulted in ER stress and persistent activation of the UPR response, leading to cellular malfunction and death^30,43,44^. In this regard, another probable reason for the HFD-induced ER stress in the current study was a relatively high proportion of palmitate (SFA) in the diet. ER stress increases the GSSG/GSH ratio and leading to increased ER calcium leakage followed by the calcium influx into the mitochondria and increased ROS production^13,45,46^. As a result, in the present study, the increased ER stress markers could be the cause of oxidative stress induction. Furthermore, the elevated plasma corticosterone concentration might be another cause of oxidative stress induction^47–49^, in the HFD group. The animal and human researches have suggested a connection between X-box binding protein 1 (XBP-1) gene expression and increased glucocorticoid levels^50,51^. Since XBP-1 is a key component of UPR, we may also conclude that HFD-induced ER stress resulted in higher plasma corticosterone levels in the HFD group. Besides, regarding the mutual interaction between oxidative and ER stress^46,52^, the induced oxidative stress, in turn, could intensify the ER stress. In the HFD group following administration of 4-PBA, as an ER stress inhibitor, reduction of the expression of BIP and CHOP proteins in pancreatic extracted ER and pancreatic tissue confirmed the involvement of HFD in the induction of ER stress^39,53^. The restoration of oxidative stress markers and plasma corticosterone level in the HFD animals, following 4-PBA injection, may also confirm the HFD-induced ER stress involvement in the alteration of these variables.

In the current study, long-term HFD consumption in the animals of HFD group increased the pancreatic *Wfs1* gene expression, while decreased its protein level. However, WFS1 protein expression was demonstrated to be increased in the extracted ER of the pancreas. It is revealed that WFS1 (an ER-resident transmembrane protein) expression increases in the presence of ER stress and contributes to the inhibition of its signaling and the associated apoptosis^14,49,54^. In the present study, the increased WFS1 protein level, along with the markers of the ER stress in the pancreatic extracted ER, may indicate a role for this protein in the preservation of ER homeostasis. Furthermore, WFS1 is a trafficking protein that binds to ER vesicles, containing proinsulin, to transfer these vesicles to Golgi complex for subsequent proinsulin processing^55^. Since, following ER stress occurrence the UPR activation limits the protein synthesis^56,57^(e.g. insulin), to adjust the protein folding ability of the ER, the WFS1 trafficking and hence its level in the cytoplasm would be reduced. Therefore, in the HFD group, the reduced level of WFS1 protein in the pancreatic tissue might be related to the decreased WFS1 trafficking. Administration of 4-PBA restored the HFD-induced alterations in the WFS1 expression levels. It should be mentioned that, there are limited studies on this subject, and the majority of the studies have been conducted on animals having a *Wfs1* gene mutation. For example, in a mouse insulinoma cell and in the stem cells of individuals both having mutant *Wfs1* gene, administration of 4-PBA decreased the levels of UPR components and suppressed the ER stress^58,59^ and increased the insulin content in these cells^59^. Given that 4-PBA inhibits the ER stress by lowering the expression of UPR components, it might regulate the WFS1 expression through the same mechanism.

According to findings of this study, HFD increased the fasting plasma glucose concentration and decreased the fasting plasma insulin concentration along with HOMA-β%. During the OGTT test, glucose tolerance was also impaired. In line with these findings, feeding HFD containing 40% lard for 8^60^ and 21 weeks^61^ in 6-week-old and 4-week-old male Wistar rats elevated the blood glucose level and reduced the blood insulin concentration, while it increased the sympathetic tone and the norepinephrine level in their pancreas, liver, and brain. According to the mentioned studies, an increase in the sympathetic tone and norepinephrine release (an inhibitor of the insulin secretion from β cells) from sympathetic terminals in the pancreatic tissue, which might be the result of increased plasma leptin level^62^, could be one of the explanations for the decrease in the plasma insulin concentration in HFD groups^60,63,64^. Furthermore, the increased plasma corticosterone level following chronic consumption of HFD, through induction of pancreatic oxidative and ER stress^65^, could reduce the plasma insulin level and caused glucose intolerance^66–69^. However, it has been shown that consumption of HFD containing lard, in Wistar rats^70,71^, and different kinds of fats (including palm, lard, and soybean oil), in mice^72^, increased plasma insulin concentration, along with an impaired glucose tolerance. Differences in the type, percentage, and/or duration of fat consumption and the species studied may explain the differences in the results. Based on the results of the abovementioned studies, in the current study, the increased plasma corticosterone and leptin concentrations, may be attributed to the decreased plasma insulin levels and impaired glucose tolerance. In this context, the HOMA-β% findings revealed the beta cell dysfunction in HFD groups, which might explain the reduction in insulin secretion during OGTT. The restoration of HFD-induced adverse changes and ultimately improved β cell function and glucose tolerance after administration of 4-PBA may emphasize the role of the ER stress in the development of these impairments.

In line with our in vivo study (OGTT) results, in the in vitro study, a chronic HFD decreased pancreatic isolated islets’ insulin content and secretion in response to basal (5.6 mM) and high (16.7 mM) glucose concentrations. Feeding a HFD^10^, lipid infusion^73^ or pancreatic isolated islets exposure to SFAs^74^ decreased insulin secretion and insulin content in response to glucose, being consistent with the findings of the current study. Accordingly, chronic exposure to high levels of fatty acids, specially SFAs, due to dietary fat, could lead to production of toxic lipids (diacylglycerol and ceramides), which cause oxidative and endoplasmic reticulum stress, mitochondrial dysfunction, and inflammation, mostly in skeletal muscle and adipocytes as well as in β cells. These conditions could be resulted in decreased insulin biosynthesis and beta cells glucose sensitivity and finally may lead to beta cells dysfunction^75^, consequently, the basal and/or stimulated-insulin secretion could adversely be affected. Moreover, Zambrano et al. found that the rat offspring exposure to the maternal HFD during developmental (pregnancy-lactation) period of body organs including pancreas, decreased basal insulin secretion from pancreatic isolated islets of offspring at PND110 in response to 5 mM glucose concentration. This may reflect the programming effect of the diet on glucose sensing machinery and/or insulin secretory pathway^76^. In addition, as mentioned above, the HFD-induced ER stress could reduce WFS1 trafficking, therefore negatively affects proinsulin processing and insulin biosynthesis, which in turn may lead to decreased insulin section. Moreover, there is an interaction between WFS1 and sarcoendoplasmic reticulum calcium ATPase (SERCA) pump, so that WFS1 affects SERCA expression level to regulate glucose-induced rise in cytosolic calcium^14^. Interestingly, ER stress impairs the interaction between this protein (WFS1) and the SERCA pump to regulate ER calcium homeostasis^54,77^. Therefore, in the presence of ER stress, WFS1 could play a role in the impairment of calcium-dependent glucose-stimulated insulin synthesis and secretion^14^. Accordingly, WFS1 depletion resulted in the impairment of pancreatic islets GSIS and insulin content^14,78^, beta-cell loss, and finally led to insulin-deficient diabetes^11,54^. In the present study, besides that the ER stress- induced changes in WFS1 expression, was involved in the decreased insulin content and secretion of the pancreatic isolated islets, considering that the development of pancreatic tissue continues about one month after birth^79^, the fatty acids present in the HFD and the increased corticosterone during this period might adversely program factors involved in insulin synthesis and secretion (e.g. glucose transporter-2 (GLUT-2), and glucokinase) in the pancreatic β cells^80,81^. On the other hand, the present study showed that the weight of body organs such as kidneys, spleen, thymus, liver, pancreas and adrenal glands increased in the HFD group. Fat accumulation in these tissues is one of the important reasons for their weight gain^82–86^. The possible reason for the increase in the weight of adrenal glands is the increase in the number and size of the cells due to the increase in the activity of the HPA axis^83^. The increased spleen weight could indicate the presence of chronic inflammation, which was induced by the elevated adipose tissue mass, following chronic HFD feeding^87^, and might be considered as another cause of decreased pancreatic insulin secretion and plasma insulin level in high-fat fed groups^88,89^. In the present study, administration of 4-PBA improved the insulin secretion and content of pancreatic isolated islets in HFD groups. In this respect, according to limited relevant researches, administration of 4-PBA, along with palmitate in culture medium of INS-1 cells or beta cells, inhibited the ER stress through decreased phosphorylated eIF2α, resulting in improved GSIS^32^. In the current study, it appears that 4-PBA administration, by reducing the expression of UPR components (Bip and Chop) and suppressing the ER stress, restored plasma corticosterone and leptin levels as well as the pancreatic WFS1 expression, which was followed by enhanced pancreatic islets’ insulin secretion and content.

## Conclusion

**Figure 10:**
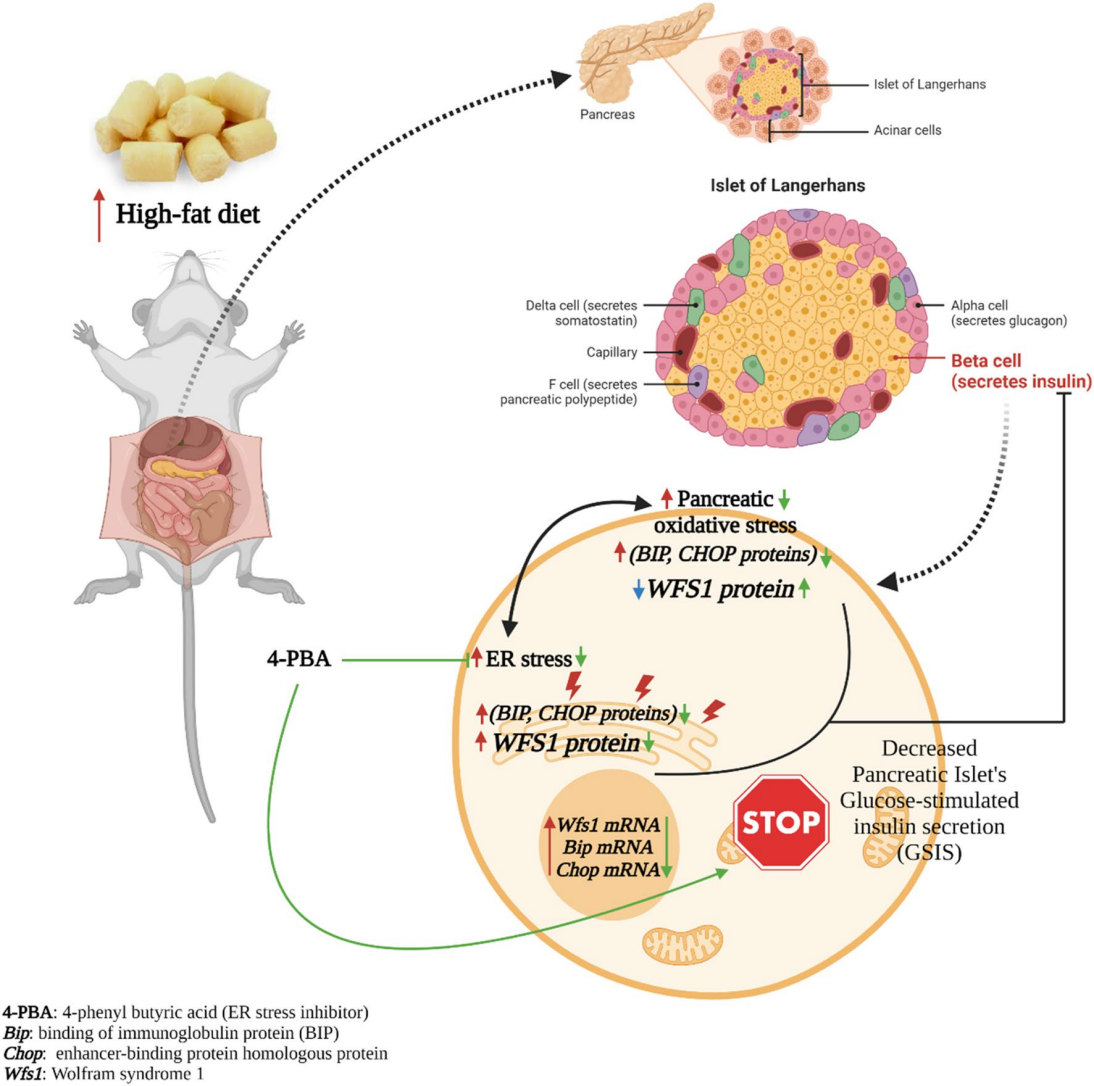
Summary of the conclusion. Regarding the present results, a long-term consumption of HFD induces oxidative and ER stress in pancreas, including the islets’ beta cells. Thus, it increases *Bip*, *Chop *and *Wfs1 *mRNA levels in the pancreatic β cells, which leads to the elevated BIP and CHOP protein amounts in the ER of these cells. Considering the role of WFS1 (a UPR component) in the maintenance of ER homeostasis, the WFS1 protein (the ER resident protein) level also increases in the ER of pancreatic β cells to restore ER homeostasis. Consequently, other than in the ER, WFS1 protein level decreases in these cells and regarding WFS1 role in insulin biosynthesis and secretion, its reduction would be resulted in the islets’ GSIS and insulin content decrement. Administration of 4-PBA restores all the changes, thus stops the pancreatic islets’ impaired GSIS and insulin content. *HFD* high-fat diet, *ER* endoplasmic reticulum, *UPR* Unfolded protein response.

In conclusion, the findings of this study suggest that long-term consumption of HFD containing 31% cow butter, increased the levels of BIP, CHOP and WFS1 proteins in the pancreatic extracted ER, reflecting the induction of ER stress, through which interfered with the contribution of WFS1 in pancreatic isolated islets’ GSIS. This intervention along with elevated plasma corticosterone and leptin levels might be the cause of the decreased biosynthesis and secretion of insulin (Fig. 10). Therefore, in the prevention and treatment of glucose metabolism disorders administrating ER stress inhibitors could be considered.

### Limitation of the study

Based on the limitations of the present study the following suggestions are proposed.:

Study the effect of HFD on the epigenetic alterations of glucose transporters, glucokinase, SERCA pump and ATP-sensitive potassium channels expression levels; and the changes of UPR components expression level along with inflammatory and apoptotic markers in the pancreatic islets of adult male rats.

## Materials and methods

### The study protocol

The methods of the present study has been reported in accordance with ARRIVE guidelines^90^. All surgical and experimental procedures were carried out with the approval of the Ethics Committee of Shahid Beheshti University of Medical Sciences, Tehran, Iran (IR.SBMU.SM.REC.1395.368) and in accordance with the guidelines for the care and use of laboratory animals (National Institutes of Health Publication No. 80–23, revised 1996). Wistar rats (Pasture Institute, Iran), both female (200 ± 30 g) and male (250 ± 50 g), were utilized in this study. The animals were kept in a light–dark cycle (12 h of light, 12 h of dark) with a temperature of 25 ± 2 °C, and they had free access to water and food. For mating, the animals were housed in a cage (two females and one male). After confirmation of pregnancy, by observing sperm in the vaginal smear, the male was removed from the cage, and the female was kept alone. After delivery, the rat pups were culled to 6–8 per litter, and the dams were cared for until the end of weaning. This study was conducted in male offspring, because females show more resistance to metabolic changes caused by HFD, and on the other hand, estradiol (females’ sex hormone) increases HPA axis activity^91^. Therefore, this study examined male rat offspring to determine the glucose metabolism impairment due to HFD; however, the findings would be considered as a basis for future studies on female offspring. After weaning, the male offspring (initial weight = 40–50 g) were randomly divided into 6 groups (6 rats/group, 6 litters/group) based on the type of diet (ND and HFD) and drug (4-PBA or DMSO). To make a high-fat diet, standard pellets were grinned and mixed with cow butter (31%), soy protein (4%), minerals and vitamins (0.7%).The combination was subsequently pelletized and given to the animals of HFD groups. After a 20-week diet (final weight of ND animals = 344 ± 6 g and HFD animals = 482 ± 10 g), 4-PBA (50 mg/kg, P21005, Sigma Aldrich, Germany) or DMSO (1.02952.1000, Merck, Germany) were IP administered twice a day for three consecutive days (10 am to 1 pm)^92^.

The study groups were as follows:

1. ND group: The rats that were fed on a normal diet from the end of weaning for 20 weeks, then the injection needle was injected, twice a day for three consecutive days without 4-PBA or DMSO.
2. ND + 4-PBA group: The rats that were fed on a normal diet from the end of weaning for 20 weeks and then received 4-PBA, twice a day for three consecutive days.
3. ND + DMSO group: The rats that were fed on a normal diet from the end of weaning for 20 weeks and then received DMSO, twice a day for three consecutive days.
4. HFD group: The rats that were fed on a high-fat diet from the end of weaning for 20 weeks, then the injection needle was injected, twice a day for three consecutive days without 4-PBA or DMSO.
5. HFD + 4-PBA group: The rats that were fed on a high-fat diet from the end of weaning for 20 weeks and then received 4-PBA twice a day for three consecutive days.
6. HFD + DMSO group: The rats that were fed on a high-fat diet from the end of weaning for 20 weeks and then received DMSO twice a day for three consecutive days.

### Normal and high-fat diet preparation

Standard chow (Behparvar animal feed producing Company, Iran) was used as a ND, with fat accounting for 11.17 percent of the total energy. To make a HFD, the following substances were added to the grinded standard pellets: cow butter (31%), soy protein (4%), mineral mixture (0.7%)^93^. Then, the mixture was grinded and formed into pellets and kept in the refrigerator (4 °C) for 3–4 days. Every day, some pellets were taken from the refrigerator and given to the animals. At the end of weaning, the animals in HFD and ND groups were fed on high-fat and normal diets for 20 weeks, respectively. Subsequently, as the animals were under their respective diet, they were injected with 4-PBA or DMSO. Table 1 illustrates the composition of normal and high-fat diets, the way of measuring the diets’ composition is explained in the Supplementary materials and methods. Table 2 shows the fatty acids profile in cow butter, and Table 3 indicates the proportion of fatty acids in the diets. The HFD had 65.06% saturated fatty acids and 33.43% unsaturated fatty acids; however, the ND had 18.53% saturated fatty acids and 81.38% unsaturated fatty acids.

**Table 1:** Composition of normal diet and high fat diet.

	Normal diet		High-fat diet	
	g%	Kcal%	g%	Kcal%
Protein	23	25.38	19.76	14.96
Carbohydrate	57.5	63.45	39.62	30
Soybean oil	4.5	11.17	1.3	2.22
Animal butter	–	–	31	52.82
Fiber	3	–	1.62	–
Ash	8		3.2	
Total phosphate	0.59	–	0.24	–
Total calcium	0.95	–	0.8	–
Mineral mixture	2.46		2.46	
Caloric density (Kcal/g)	3.63		5.28

**Table 2:** Profile of fatty acids in cow butter.

	Common name	Percentage of fatty acid
C4:0	Butyric acid	1.05
C6:0	Caproic acid	0.8
C8:0	Caprylic acid	0.6
C10:0	Capric acid	2.04
C12:0	Lauric acid	3.2
C14:0	Myristic acid	12.3
C14:1c n-5	Myristoleic acid	1.1
C16:0	Palmitic acid	38.3
C16:1c n-7	Palmitoleic acid	0.7
C18:0	Steric acid	6.8
C18:1c n-9	Oleic acid	30.5
C18:2c n-6	Linoleic acid	2.2
C20:0	Arachidonic acid	0.2

**Table 3:** Percentage of fatty acids in normal and high-fat diets.

	Common name	Percentage of fatty acid	
		ND	HFD
C4:0	Butyric acid	0	0.9
C6:0	Caproic acid	0	0.8
C8:0	Caprylic acid	0	0.6
C10:0	Capric acid	0	2.25
C12:0	Lauric acid	0.3	4.21
C14:0	Myristic acid	0.27	11.74
C14:1c n-5	Myristoleic acid	0	1.19
C16:0	Palmitic acid	14.4	40.7
C16:1c n-7	Palmitoleic acid	0	0.4
C18:0	Steric acid	3.25	3.06
C18:1c n-9	Oleic acid	32.34	26.9
C18:2c n-6	Linoleic acid	44.97	4.34
C20:0	Arachidonic acid	0.11	0.3

### Blood sampling and HOMA-β% calculation

In all groups (36 rats, 6 rats per group), fasting (14–16 h, 8–8:30 a.m.) blood sampling was carried out by tail cut under isoflurane (Baxter, USA) anesthesia (6.5 mL/L/kg of isoflurane/desiccator volume/rat body weight). This anesthetic agent has the least effect on plasma metabolic parameters^94^. Blood samples were collected in 1.5 mL Eppendorf tubes containing 10 µL/mL heparin (5000 IU/mL, Caspian Tamin, Rasht, Iran) and centrifuged at 664×*g* for 10 min. Subsequently, plasma was removed and kept at − 70 °C until glucose, insulin, leptin, and corticosterone levels were measured.

To assess the β cells function, HOMA-β% index was calculated using the following formula^95^: HOMA-β% = (20 × fasting insulin (µU/mL)/fasting glucose (mM)-3.5).

### Oral glucose tolerance test (OGTT)

In animals of all groups (36 rats, 6 rats per group), after fasting blood sampling (0 min), glucose (Merck, Germany) was given via oral gavage as a 45% solution at a rate of 2 g/kg body weight^96^. Then, blood samples were taken to measure glucose and insulin concentrations 30 and 120 min after glucose ingestion, (it is noteworthy that in this test to determine glucose intolerance, fasting and 2 h blood sampling are essential^97^, moreover since after glucose loading, according to the previous studies^36,96,98^, which in addition to 30 min the time points of 60 min and 90 min were also assessed, the plasma glucose level has been peaked at 30 min, thus blood sampling at this time could increase the accuracy of the interpretation^97^).

All samples were centrifuged (664**×g**, Sigma, Germany) for 10 min at 4 °C. Then plasma was removed and kept at − 70 °C to determine glucose and insulin concentrations.

### The assessment of plasma parameters

Plasma glucose was measured using glucose oxidase method (Pars Azmoon Co., Tehran, Iran, sensitivity 5 mg/dL). Rat insulin ELISA Kit (Mercodia, Sweden, sensitivity 0.07 µg/L), rat leptin ELISA Kit (ZellBio GmbH, Ulm, Germany, sensitivity 0.05 ng/mL), and rat corticosterone ELISA Kit (ZellBio GmbH, Ulm, Germany, sensitivity 1.6 nmol/L) were used to determine plasma insulin, leptin and corticosterone concentrations, respectively. The intra-assay coefficients of variation for the plasma glucose, insulin, leptin and corticosterone measurements were 1.28, 3.1, 5.2 and 4.1% respectively.

### Tissue isolation

One day after the end of 20 weeks of diet, following 12–14 h fasting and under isoflurane anesthesia, the animals (6 rats per group, 6 litters/group) were decapitated and dissected, after draining the trunk blood. Then their pancreas, kidneys, spleen, thymus gland, adrenal glands, and liver, as well as intraabdominal, mesenteric and retroperitoneal fat were quickly removed and weighed after rinsing with normal saline.

### Assessment of pancreatic MDA and GSH levels and catalase activity

For pancreatic tissue preparation, the animal’s isolated pancreas (6 rats/group, 6 litters/group) was cut into several pieces and homogenized by a lab homogenizer (TOMY Micro Smash MS 100, Indonesia) in a lysis buffer [containing Tric-HCl pH = 8, sodium deoxycholate, NaCl, sodium dodecyi sulfate (SDS), ethylenediamine tetra-acetic acid (EDTA), Triton and H_2_O]. Then the homogenate was centrifuged (at 12,000 rpm for 30 min) and the supernatant was collected to quantify its total protein concentration, using the Bradford method, and to perform the assessments.^99^

The pancreatic MDA level, as a biomarker of lipid peroxidation^100^, was determined using a commercial colorimetric kit (Zellbio, Germany, sensitivity 0.1 µM). The level of this oxidative stress marker was reported in mg of protein.

To assess the pancreatic GSH content the Ellman method^101^ was used. Briefly, a solution was made by adding a phosphate buffered saline (PBS) to the pancreatic homogenate containing 120 μg protein, then trichloroacetic acid was added and centrifuged. The prepared supernatant was then incubated with DTNB [5,5-dithiobis- 2-nitrobenzoic acid (2 mg/mL PBS)] (D218200, Sigma, USA) for 10 min at 37 °C. Finally, at a wavelength of 405 nm, the absorption rate was measured using an ELISA reader (BioTeK, ELX800TS, USA). The GSH level was expressed as μmol per mg of protein.

Pancreatic catalase activity was evaluated using the Goth method^102^, accordingly PBS was added to the supernatant containing 180 μg protein. Then, the solution was incubated with H_2_O_2_ (0.01 M) at 25 °C for 15 min. Subsequently, ammonium molybdate (6.35 mg/mL PBS) (277908, Sigma, USA) was added to stop the enzymatic reaction. The degradation rate of H_2_O_2_ was indicated by measuring the absorption rate at a wavelength of 405 nm. The results were expressed as µmol H_2_O_2_/min/mg protein.

### Real-time quantitative reverse transcription PCR (qRT-PCR)

To determine the relative expression of *Wfs1*, *Bip*, and *Chop* mRNA levels, 50 mg of the pancreatic tissue (6 rats per group, 6 litters/group) was isolated and collected in RNAse-free microtubes containing RNA stabilizer (RNA_later_, QIAGEN Company, Germany). These samples were sent to the Laboratory of Endocrinology and Metabolism Research Institute of Shahid Beheshti University of Medical Sciences, Tehran, Iran, for RNA extraction. RNA was extracted from pancreatic tissue samples according to the manufacturer’s instruction of the TRI _zol_ reagent (Invitrogen u.s.cat. no.15596-026). According to the NanoDrop spectrometer (Thermo Fisher Scientific, Waltham, USA) and the absorption ratio of 260/280 nm, the quality and amount of the RNA from all samples were in an acceptable range. To remove any potential DNA contamination and improve the sample quality, the total RNA was processed with DNase I enzyme, before production of complementary DNA (cDNA). The single-stranded cDNA molecule was synthesized using a Fermentas kit (Thermo Scientific, USA). The synthesized solution was kept at – 20 °C. The appropriate primers for the target genes were designed using the gene bank data from the National Center for Biotechnology Information (NCBI). For *Wfs1*, *Bip*, and *Chop* genes expression analysis, the GAPDH gene was chosen as the housekeeping gene. Table 4 presents the sequences of the primer used in RT-PCR. The qPCR, using a Real-Time PCR instrument (Rotor-Gene 6000, Sydney, Australia), was performed in 25 μL volumes containing 12.5 μL 2× SYBR Green Master mix (Thermo Scientific, USA), 1 μL forward primers, 1 μL reverse primers, 7.5 μL RNase-free water, and 3 μL of cDNA Initial denaturation (10 min at 95 °C) was followed by 40 thermal cycles of 15 s at 95 °C, 45 s at 60 °C, and 40 s at 72 °C. Fluorescence activity was used to assess the real-time quantification. The difference between the threshold cycle (CT) of the target gene and the CT of the GAPDH gene was used to determine the *Wfs1*, *Bip*, and *Chop* mRNA levels in each sample using the following formula^103,104^:

$$\Delta {\text{C t }} = {\text{ CT }}\left( {{\text{target}}} \right) \, {-}{\text{ CT }}\left( {{\text{reference}}} \right).$$

**Table 4:** Primers used for real-time PCR analysis.

Primer name	Gene bank accession no.	Primer sequence (5^0^ → 3′)
WFS1	MW_6446.2	Forward: GCCCTGGTCATGTACTGGAAA
		Reverse: CCCTCCATCCTGTTCGTTGA
Bip	NM_013083.2	Forward: CCTGCGTCGGTGTGTTCAAG
		Reverse: AAGGGTCATTCCAAGTGCG
CHOP	NM_001109986.1	Forward: GAAAGCAGAAACCGGTCCAAT
		Reverse: GGATGAGATATAGGTGCCCCC
GAPDH	MW_5994.9	Forward: GACAGCCGCATCTTCTTGTG
		Reverse: AGAGAAGGCAGCCCTGGTAA

### Pancreatic rough endoplasmic reticulum (RER) isolation

In this experiment, the rough vesicles derived from the RER of rat whole pancreatic cells were prepared. Briefly, 10 adult male rats (10 rats per group, 10 litters/group) were anesthetized by isoflurane and euthanized by decapitation, and then their pancreases were removed and immediately put in a beaker containing 30 mL of ice-cold (0.25 M) sucrose solution to be rinsed. Afterward, the pancreases were cut into very tiny pieces. The pancreatic tissues were homogenized using a potter homogenizer (Potter–Elvehjem Homogenizer, Iran), at 2850 rpm. After adding 60 mL of the ice-cold sucrose solution, the homogenate was filtered and transferred into a falcon tube, then centrifuged for 13 min at 4000 rpm. Afterward the supernatant was used in stage 2 (to extract RER vesicles) and the precipitate (containing cytoplasmic components and membrane proteins) was used for determining WFS1, BIP, and CHOP proteins by a western blot method (Stage 1). Thereafter, the supernatant was centrifuged at 35,800 rpm for 67 min, at 4 °C (Beckman model J-21B, USA) (Stage 2). After dissolving the pellet in 10 mL of ice-cold sucrose (2 M), the solution was transferred to a 20 mL glass homogenizer, and was manually homogenized for 20–25 times to obtain a homogenous suspension. The suspension was subsequently centrifuged at 67,000 rpm for 75 min at 4 °C in a sucrose gradient condition (including 1 M and 2 M sucrose solution) (Stage 3). Additionally, the resulting pellet was dissolved in 20 mL of purification solution (including sucrose 0.25 mM, imidazole 3 mM, Na pyrophosphate 0.5 mM) and centrifuged three times at at 40,400 rpm for 47 min at 4 °C (Stage 4). The resulting pellet (containing RER vesicles) was dissolved in 2 ml sucrose (0.25 mM) and imidazole (3 mM), at 7 mg/mL concentration and stored in microtubes (10 µL aliquots) at − 70 °C and used for WFS1, BIP, and CHOP proteins determination by a western blot method^105,106^.

### Western blotting

In the first step, the protein concentrations of the above prepared samples [i.e. the extracted ER and the precipitate (containing cytoplasmic components and membrane proteins)] were measured using the Bradford protocol^107^. Then, proteins (60 µg) from each fraction were loaded onto SDS-PAGE (12% Bis–Tris Plus gels). After wet electrophoretic, the samples were transferred onto a Polyvinylidene Difluoride (PVDF) membrane, and then blocked in a Tris-buffered saline (TBS) containing 0.1% Tween 20 (v/v) and 5% (w/v) bovine serum albumin (BSA) for 2 h, at room temperature. Then, membranes were soaked overnight at 4 °C in primary antibodies (including WFS1 # STJ26110, St John’s Laboratory; BIP # ab227865, Abcam; and CHOP # M00311, Boster Bio), which were diluted in PBS + 0.1% Tween 20 + 3% BSA. After three steps of washing, membranes with antibodies were soaked in a secondary antibody (Anti-Rabbit IgG # CS7074, Cell Signaling), which were diluted in PBS + 0.1% Tween 20 + 3% BSA for 1 h at room temperature. Finally, the membranes were treated with an ECL kit (Amersham ECL select™, GE Healthcare, USA) for chemiluminescence detection according to the manufacturer’s instructions. To quantify the bands, densitometry of protein bands was performed using image J software. As positive controls, calnexin (# SC-46669, Santa Cruz), an ER marker, and β-actin (# ab8227, Abcam) antibodies were used^108^.

### Pancreatic islets isolation

Islet isolation was performed according to the collagenase technique of Lacy and Kostianovsky^109^ with slight modifications. Following an overnight fasting (12–14 h), the animals (4 rats/group, 4 litters/group) were anesthetized (using isoflurane) and decapitated. Their trunk blood was then drained and the abdomen was opened, subsequently the entrance of the common bile duct to the duodenum was blocked with a forceps. Following the insertion of a catheter (Portex Intravenous Cannula 2.5 F, 0.75 mm OD) into the common bile duct, 10 mL of the cold Hanks saline solution (containing in mM: NaC1, 137; KCI, 5.4; CaCl_2_, 1.2; MgSO_4_7H_2_O, 0.8; Na_2_HPO_4_·2H_2_O, 0.3; KH_2_PO_4_, 0.4; NaHCO_3_, 4.2 (Merck, Germany) containing 0.75 mg/mL collagenase P (Roche, Cat. # 11213 865 001, Germany) was injected into the pancreas. Following the injection, the dilated pancreas was initially removed and placed in a petri dish, and its adipose tissue and lymph nodes were removed, afterward the sample was transferred to a 50 mL falcon tube and incubated at 37 °C for 17 min in a water bath. Afterward, 35 mL of cold Hanks solution was added to the falcon to stop digestion and following one minute of shaking, its content was transferred to a 150 mL crystallizer. Then, cold Hanks solution was added to the edge of the crystallizer and mixed gently by a plastic pipette (movette 5 mL (PK40) 005-40673A, Laborimpex, Belgium), when the islets had settled, the top solution was aspirated using a suction system, then the aspirated volume was replaced with cold Hanks solution (this washing process was repeated two more times). Finally, after the last washing step the isolated islets were handpicked (first picking) under a stereomicroscope (Blue Light stereomicroscope, USA).

### Evaluation of the isolated islets’ GSIS

At this point, for each rat in the study groups, the handpicked islets (second picking) were transferred to plastic cups (GAP-01 GAP PS, Laborimpex, Belgium) (10 islets per cup; 2 cups per each concentration of 5.6 and 16.7 mM glucose per rat), so that there were 8 cups/glucose concentration/group. Using a stereomicroscope, the excess Hanks solution in each cup was removed as much as possible, and 1 mL of the Krebs solution (pH 7.4) [containing in mM: NaCl, 111; KCl, 5; MgCl_2_ 6H_2_O, 1; CaCl_2_, 1; NaHCO_3_, 24 (Merck, Germany); Hepes, 10 (Sigma, USA) and BSA, 0.5 g/dL (Sigma, USA)] containing 5.6 or 16.7 mM glucose was immediately added to each cup. All procedures for islets isolation were carried out on the ice tray. The cups were covered and incubated for 90 min (at the beginning, the cups were gassed with 95% O_2_/5% CO_2_ for 5 min) at 37 °C in a water bath. Then, the supernatant was removed and stored at − 70 °C for insulin assay^2,110^.

### Evaluation of the isolated islets’ insulin content

To measure the islets insulin content, after the removal of supernatant from the aforementioned cups, 1 mL of ethanol acid solution (0.18 M-HCl in 70% ethanol) was added to the islets left in the cups. The islets were then kept overnight at 4 °C. Then, the solution was centrifuged for 10 min at 1300×*g* to remove cell debris, and the supernatant was used to assess the islets total protein (using Bradford protocol^107^) and insulin levels^2,110^.

To assess the insulin secretion and content of the isolated islets, a rat insulin ELISA Kit (Mercodia, Sweden, sensitivity 0.07 µg/L) was used. The intra- and inter assay coefficients of variations for these measurements were 3.4% and 5.5%, respectively.

### Statistical analysis

The results were expressed as mean ± SEM. Graph Pad Prism version 8 and SPSS version 21 were used as statistical software programs. To compare various diets on different times, three-factor mixed-model analysis of variance (ANOVA) (regarding time as a repeated factor, and diet and drug as between subjects factors) followed by the Tukey post hoc test, was used. To compare various diets in different groups, a two-way ANOVA (regarding diet and drug as independent factors) followed by the Tukey post hoc test, was used. P < 0.05 was considered statistically significant.

## Acknowledgments

This article has been extracted from the thesis written by Mrs. Fateme Binayi in School of Medicine Shahid Beheshti University of Medical Science (Registration No: 310). This work was supported by the Neurophysiology Research Center, Shahid Beheshti University of Medical Sciences [Grant number 739].

## Supplementary Information

Supplementary Information 1.

Supplementary Information 2.

Supplementary Information 3.

## Data Availability

All data generated or analyzed during this study are included in this article. Further enquiries can be directed to the corresponding author.
