# Supplementary material for: Endoplasmic reticulum stress inhibition ameliorated WFS1 expression alterations and reduced pancreatic islets’ insulin secretion induced by high-fat diet in rats
Source: Sci Rep. 2023 Feb 1;13:1860. doi: 10.1038/s41598-023-28329-1 (PMC9892558; doi:10.1038/s41598-023-28329-1)
Supplement: Supplementary file 1 — Supplementary Information 1. [file 41598_2023_28329_MOESM1_ESM.docx]

**Supplementary Materials and Methods**

# Measurement of the contents and calories of the ND and HFD components

# In this regard, before making the HFD, the standard pellets were analyzed by a specialized food analyzing laboratory (Viromed lab Food and Beverag Manufacturing, Tehran, Iran, http://viromedlab.com). Then, considering the content of the standard pellets (shown in Table 1), to make 100 g of HFD containing 31% cow butter, 64.3 g of grinded standard pellets was mixed with 31 g cow butter, 4 g soy protein and 0.7 g mineral mixture. To measure the components of the prepared HFD, the HFD pellets were sent to the aforementioned laboratory to analyze and determine its components (shown in Table 1). Then, to calculate the percentage of calories of protein and carbohydrate in the diets (ND and HFD), the amount of each, in 100 g of the diets, was multiplied with 4 (1 g protein = 4 kcal and 1 g carbohydrate = 4 kcal) and for calculating the percentage of calorie of fat in 100 g of the diets the total amount of fat was multiplied with 9 (1 g fat = 9 kcal)^1^. Subsequently, the sum of the calories of these components (protein, carbohydrate and fat), was calculated in 100 gram of each diet and then the kcal% of each component was calculated. In this regard, the calculated Kcal of each protein, carbohydrate and fat in 100 g of the diet was divided by the sum of the calories and the result was multiplied with 100 (shown in Table 1).

1. Kwon, Y.-J., Lee, H.S., Park, J.-Y. & Lee, J.-W. Associating intake proportion of carbohydrate, fat, and protein with all-cause mortality in Korean adults. *Nutrients* **12**, 3208 (2020).
