# Supplementary material for: Endoplasmic reticulum stress inhibition ameliorated WFS1 expression alterations and reduced pancreatic islets’ insulin secretion induced by high-fat diet in rats
Source: Sci Rep. 2023 Feb 1;13:1860. doi: 10.1038/s41598-023-28329-1 (PMC9892558; doi:10.1038/s41598-023-28329-1)
Supplement: Supplementary file 2 — Supplementary Information 2. [file 41598_2023_28329_MOESM2_ESM.docx]

**
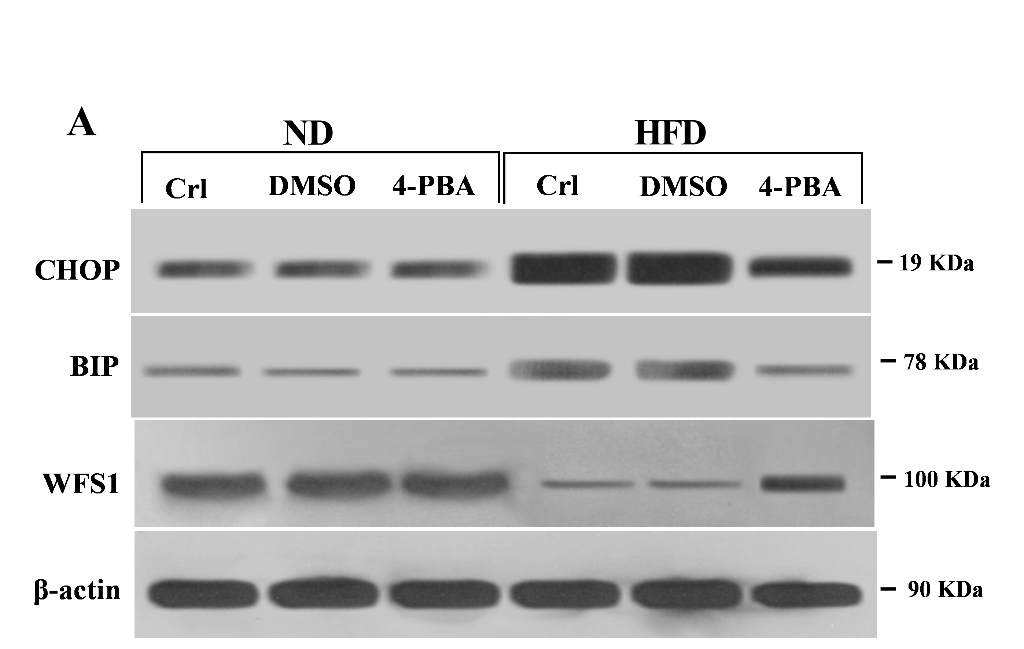
**

**
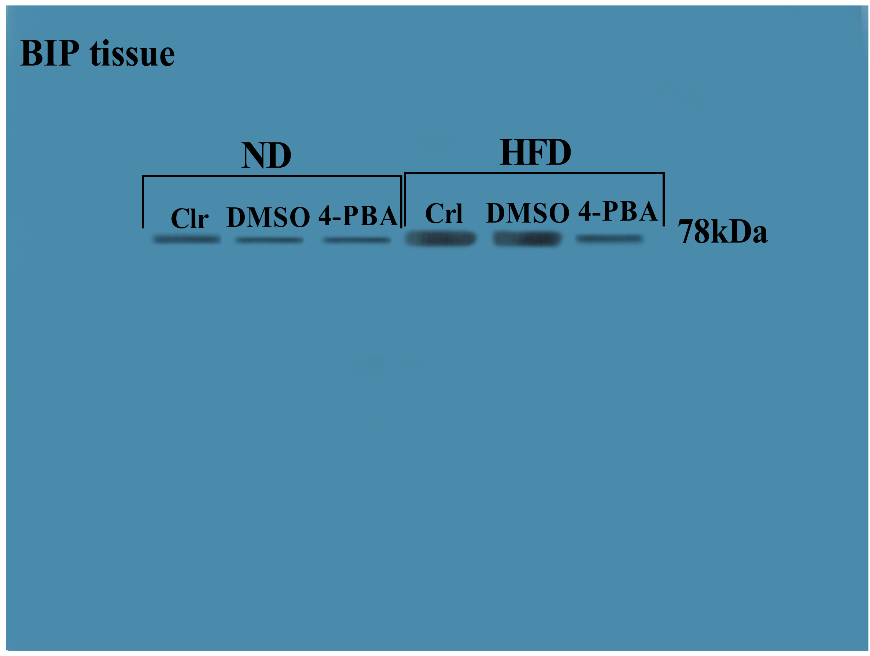

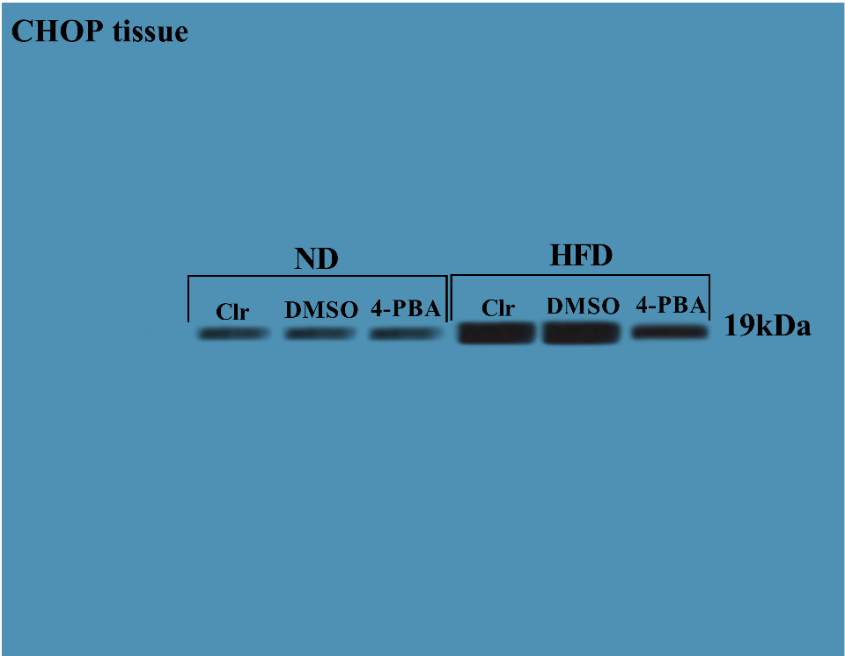
**

**
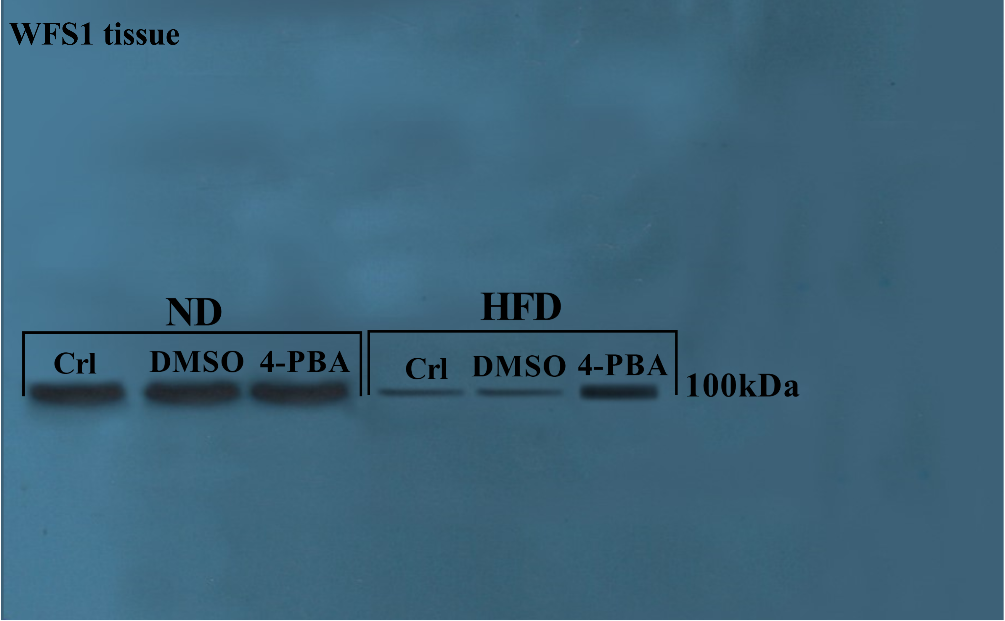
**

**
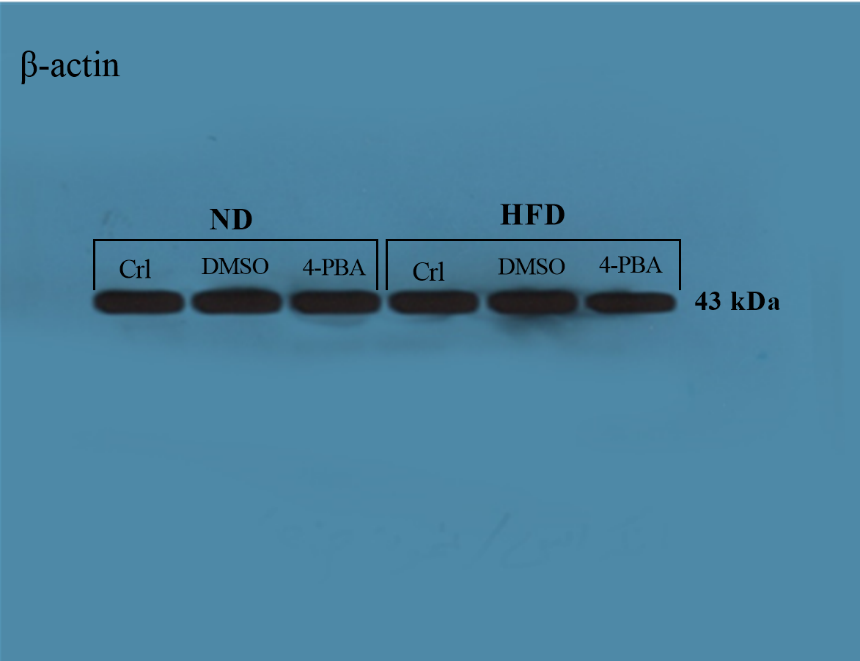
**

**Fig. 3. Effect of high-fat diet and/or 4-PBA on the (A, B) CHOP, (A, C) BIP and (A, D) WFS1 protein levels of the pancreas**
