# Supplementary material for: Endoplasmic reticulum stress inhibition ameliorated WFS1 expression alterations and reduced pancreatic islets’ insulin secretion induced by high-fat diet in rats
Source: Sci Rep. 2023 Feb 1;13:1860. doi: 10.1038/s41598-023-28329-1 (PMC9892558; doi:10.1038/s41598-023-28329-1)
Supplement: Supplementary file 3 — Supplementary Information 3. [file 41598_2023_28329_MOESM3_ESM.docx]

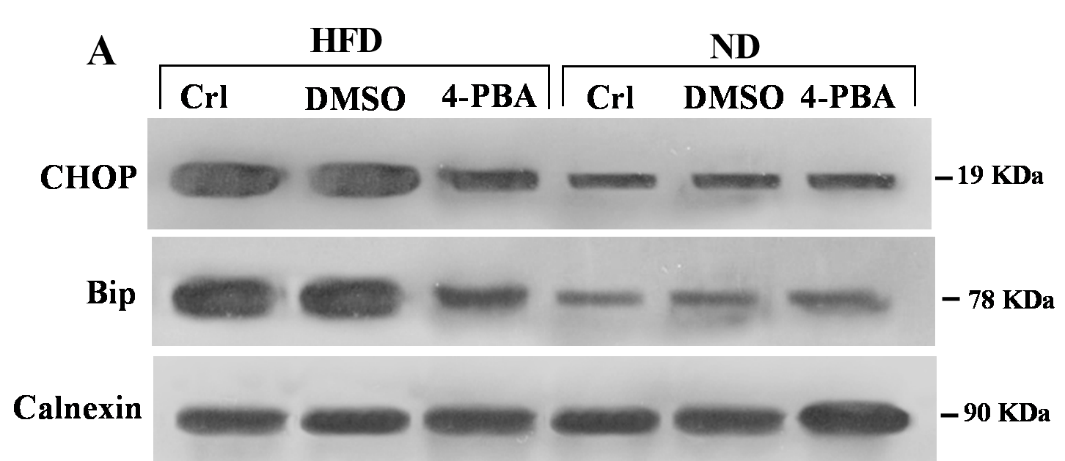


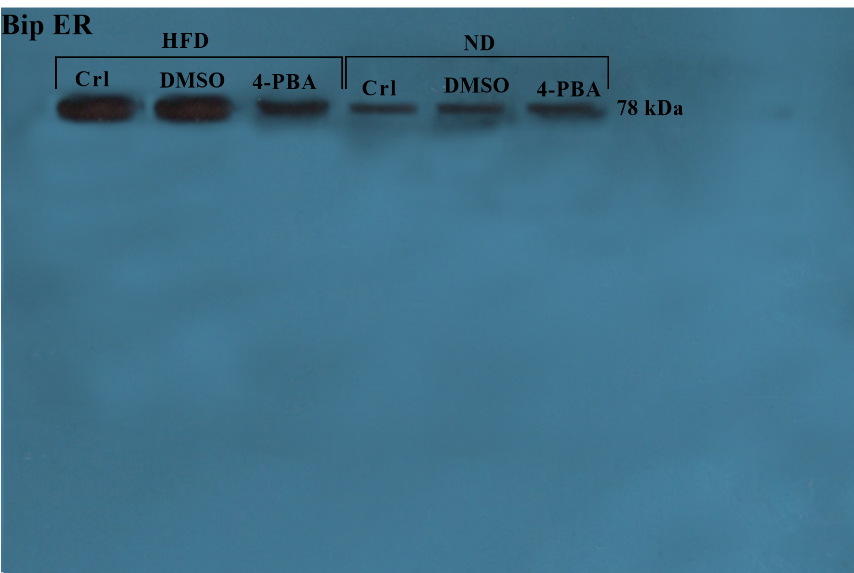

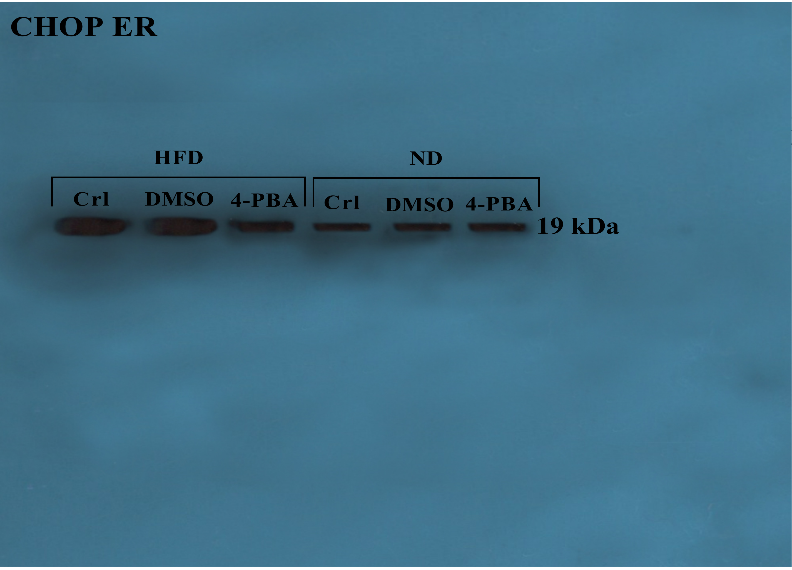


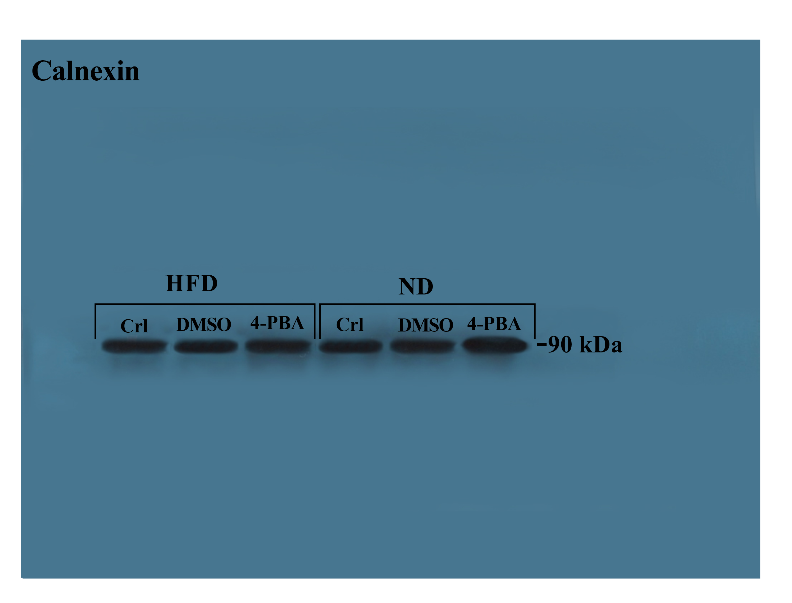


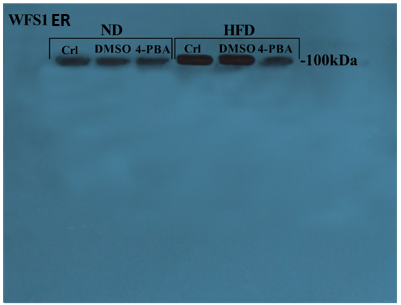

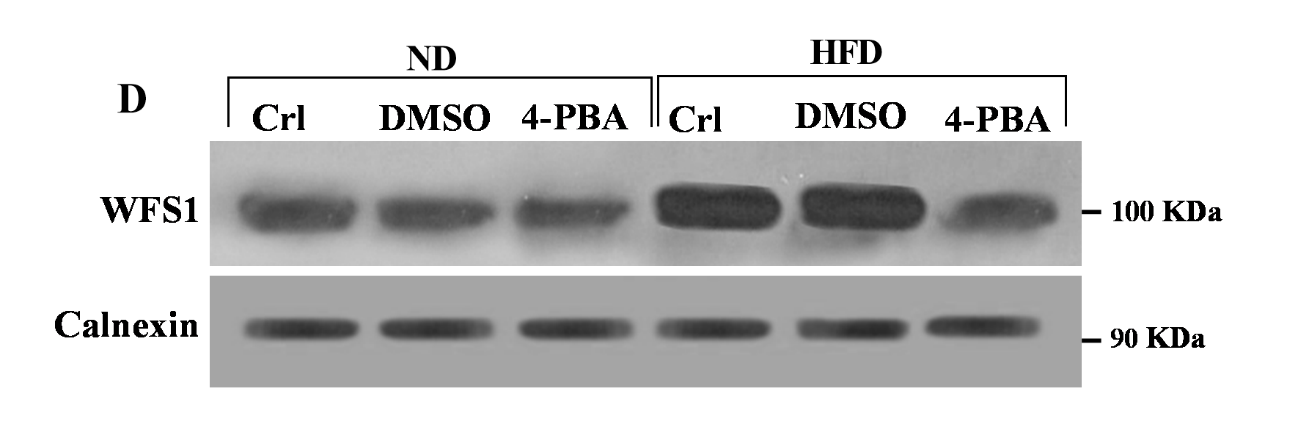


**
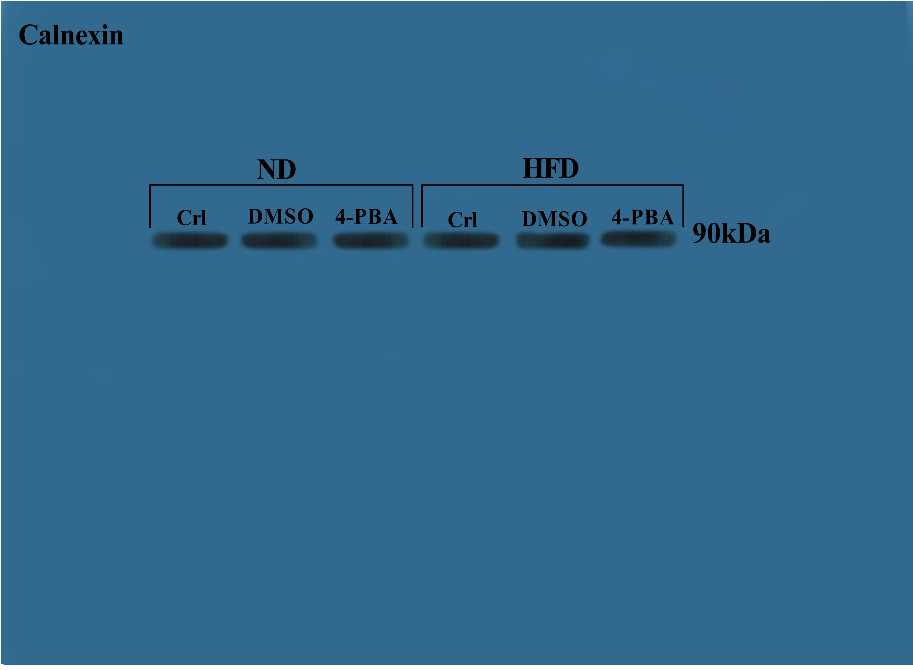
**

**Fig. 4. Effect of high-fat diet and/or 4-PBA on the (A, B) CHOP, (A, C) BIP and (D, E) WFS1 protein levels of the extracted ER of pancreas.**
